# “Help Me Control My Impulses!”: Adolescent Impulsivity and Its Negative Individual, Family, Peer, and Community Explanatory Factors

**DOI:** 10.1007/s10964-023-01837-z

**Published:** 2023-08-24

**Authors:** Célia Barreto Carvalho, Ana Moura Arroz, Raquel Martins, Rodrigo Costa, Filipa Cordeiro, Joana Moura Cabral

**Affiliations:** 1 Faculty of Social and Human Sciences, University of the Azores, Ponta Delgada, Portugal; 2 Cognitive and Behavioural Centre for Research and Intervention, Faculty of Psychology and Education Sciences, University of Coimbra, Coimbra, Portugal; 3 Centre for Ecology, Evolution and Environmental Changes, Azorean Biodiversity Group (cE3c/ABG); CHANGE – Global Change and Sustainability Institute, University of the Azores, Ponta Delgada, Portugal; 4 Gaspar Frutuoso Foundation, University of the Azores, Ponta Delgada, Portugal

**Keywords:** Adolescence, Impulsivity, Peer context, school context, family context

## Abstract

The literature shows that impulsivity, prevalent in adolescence, is negatively linked with a variety of psychosocial factors (e.g., positive interpersonal relationships, emotion regulation); however, there is limited research examining the relative contribution of multiple factors for this trait nor exploring how these factors influence the associations between impulsivity and risk-related outcomes. Drawing on multiple components of the unified theory of development (i.e., psychological variables, peers subsystem, community subsystem, family processes subsystem), this cross-sectional study aims to identify explanatory psychosocial variables (i.e., early memories of warmth and safeness, rational decision-making style, resilience, emotion regulation, coping, parental attachment, social group attachment, satisfaction with school and family-related variables) that are negatively related with impulsivity, in younger (13–15) and older (16–19 years) adolescents, and explore their moderating role in the associations between this trait and some risk-related outcomes (i.e., verbal aggression, anger, self-harm, other high-risk behaviors). A representative sample of 6894 adolescents (52.9% female) living in the Azores (Portugal), with ages ranging from 13 to 19 (*M* = 15.4), was used. Two stepwise multiple regressions, one for each age group, revealed that only emotion regulation, parental attachment, and social group attachment had a negative effect on impulsivity in both age groups; additionally, satisfaction with teachers also had this effect in younger adolescents. The first three variables weakened the positive associations between impulsivity and the risk-related outcomes. These results suggest that the psychological system and all subsystems of the social context measured play a relevant role in explaining adolescent impulsivity and that it may be reduced by promoting emotion regulation, positive parenting practices, healthier relationships with peers, and healthier relationships with teachers.

## Introduction

High levels of impulsivity have been consistently found to be relatively common in adolescence (Rosenbaum & Hartley, 2019), with this trait increasing the risk for a variety of high-risk behaviors such as delinquency (e.g., Ragan et al., 2022), aggression (e.g., Cao & An, 2020), substance use (e.g., VanderVeen et al., 2016), self-harm (e.g., Hasking & Claes, 2020), and suicidal behavior (e.g., MacPherson et al., 2022). These behaviors, which are often interrelated (Bozzini et al., 2021), increase the risk for adolescent morbidity and mortality (Kipping et al., 2012). Additionally, multiple previous studies have examined the negative associations between many biopsychosocial factors and impulsivity in adolescence, either as antecedents of this trait—therefore having a causal relationship with it—or as negative correlates. Due to the nature of these variables, they can be examined in light of the unified theory of development (Sameroff, 2010), which posits that human development is influenced by an array of interacting biological (e.g., neurophysiology, neuroendocrinology), psychological (e.g., social competence, identity), and social (e.g., family, school) factors. Even though adolescent impulsivity has a strong neurobiological component (Steinberg, 2008), the literature shows that lower levels of impulsivity/higher levels of self-control are predicted by: biological factors, namely the female gender (e.g., Perez et al., 2016) and older age groups (e.g., Inuggi et al., 2014); variables related to the family structure subsystem, including higher parental socioeconomic status (e.g., Assari et al., 2018); aspects related to the parent characteristics subsystem, namely higher parental self-control/lower parental impulsivity (e.g., Bolger et al., 2022); variables related to the peers and the community subsystems, such as positive school environment and positive peer relationships (e.g., Joo & Lee, 2020); and factors related to the family processes subsystem, including positive parenting (e.g., Khurana & Romer, 2020). Moreover, impulsivity has been found to be negatively correlated with: aspects related to the peers and the community subsystems, such as positive peer relationships (e.g., Moyano et al., 2022); variables related to the family processes subsystem, including early memories of warmth and safeness (Barreto Carvalho et al., 2015) and positive family relationships (e.g., Song et al., 2019); and psychological factors, such as rational decision-making style (Jelihovschi et al., 2018), resilience (Ran et al., 2022), problem-focused coping (Li et al., 2019), and emotion regulation (e.g., Hasking & Claes, 2020). However, no previous study, to the best of the authors’ knowledge, has examined the contribution of multiple negative correlates/protective factors for adolescent impulsivity in light of multiple subsystems of the unified theory of development (Sameroff, 2010) nor explored the moderating role of these variables in the (positive) associations between impulsivity and risk-related factors. To address this gap, the present study aims to determine an array of possible psychosocial variables (i.e., early memories of warmth and safeness, rational decision-making style, resilience, emotion regulation, coping, parental attachment, social group attachment, satisfaction with school and family-related variables) that negatively associate with adolescent impulsivity and to explore the moderating role of these variables in the positive associations between this trait and some risk-related outcomes (i.e., verbal aggression, anger, self-harm, other high-risk behaviors).

### Adolescence, Impulsivity, and High-Risk Behaviors

Adolescence is a critical developmental period ranging from 10 to 19 years old (World Health Organization [WHO], n.d.) characterized by multiple physical, neurological, psychological, emotional, and social changes (e.g., Dahl et al., 2018) associated with an array of vulnerabilities and risks (Zanus et al., 2021), namely impulsivity (Rosenbaum & Hartley, 2019), a complex construct involving difficulty controlling desires and impulses, as well as actions without a deliberate plan that are likely to result in negative consequences (e.g., Shulman et al., 2016). Two other constructs that are directly related to impulsivity are low self-control (Gottfredson & Hirschi, 1990)—characterized, among other things, by carelessness and a tendency for immediate gratification of desires and lack of planning—and sensation-seeking (Stoyanova & Ivantchev, 2021)—defined as the tendency to seek novel and highly stimulating experiences (Zuckerman, 1994), with some authors conceptualizing it as a distinct trait from impulsivity (e.g., Whiteside & Lynam, 2001). There is a vast literature showing that adolescent impulsivity is associated with a variety of high-risk behaviors—with a tendency for those who engage in any high-risk behavior to engage in others having been found (Bozzini et al., 2021)—including delinquency (e.g., Menting et al. (2015); Ragan et al., 2022), aggression (e.g., Cao & An, 2020), gambling (e.g., Liu et al., 2013), substance use (e.g., VanderVeen et al., 2016), risky sexual behavior (e.g., Hentges et al., 2018), disordered eating (Hasking & Claes, 2020), self-harm (e.g., Hasking & Claes, 2020), and suicidal behavior (e.g., MacPherson et al., 2022). Some studies have discussed the evolutionary notion that adolescents’ impulsivity—alongside other characteristics of this developmental period (e.g., importance of peers, sensation-seeking)—is related to the high flexibility of cognitive control typical of adolescence, which is useful for navigating the complex social contexts of this developmental period (Crone & Dahl, 2012); this level of adolescent cognitive control facilitates gradual exploration and autonomy (Icenogle & Cauffman, 2021; Steinberg, 2008). Moreover, on the one hand, it increases the likelihood of showing high-risk behaviors and vulnerabilities; on the other hand, it contributes to adolescents’ heightened ability to learn from experience (Crone & Dahl, 2012; Decker et al., 2015).

### Exploring Negative Correlates and Protective Factors of Adolescent Impulsivity Using the Unified Theory of Development

The unified theory of development (Sameroff, 2010), which integrates four models—the personal change model, the contextual model, the regulation model, and the representational model—posits that human development is influenced by several interacting individual and contextual factors. It presents a biopsychosocial ecological system in which the individual is composed of a cluster of interacting biological (e.g., neurophysiology, neuroendocrinology), psychological (e.g., social competence, identity), and social (e.g., family, school) processes; the first two domains of processes (i.e., biological, psychological) comprise the self-regulation system and the social factors comprise the other-regulation system. For the purposes of this study, the two models that are most relevant for explaining impulsivity are the contextual model and the regulation model. The first model (i.e., contextual) presents six subsystems that promote or limit development through risk and promotive factors: family processes (e.g., parental attachment, early memories of warmth and safeness), parent characteristics (e.g., parental impulsivity, level of education), family structure (e.g., socioeconomic status, welfare status), family management (e.g., social resources, informal network), peers (e.g., social group attachment, association with pro-social peers), and community (e.g., school satisfaction, neighborhood problems). All these contexts have an effect on primarily five domains of youth developmental outcomes including psychological adjustment, self-competence, conduct problems, extracurricular involvement, and academic performance; impulsivity is a key factor in psychological adjustment and in self-competence, and is a risk factor for conduct problems. Lastly, the regulation model emphasizes the interaction between the individual and the social contexts and explains human regulation as it develops from the primarily biological (e.g., hunger, temperature) to the psychological and social processes (e.g., impulse control, social interactions); particularly, the ability to self-regulate develops over time through social interactions, firstly with the primary caregivers and then with peers and other significant figures. The literature shows that an array of biopsychosocial factors comprised in this unified theory are linked with adolescent impulsivity, with this trait having a strong neurobiological component (Steinberg, 2008) and being influenced by multiple psychosocial factors during this developmental period. Given that this study focuses on the negative psychosocial factors that negatively associate with impulsivity in adolescence, the literature outlined below includes studies that found some variables of this nature to be negatively linked with this trait during this period, either as being antecedents of this trait—and therefore having a causal relationship with it—or as being negative correlates.

Some of the variables found to negatively associate with impulsivity are assumed to have a negative causal relationship with this trait because reverse causation is not possible, namely biological variables (e.g., gender, age), as well as variables related to the family structure subsystem (e.g., parental socioeconomic status) and to the parent characteristics subsystem (e.g., parental impulsivity). More specifically, female youth have been found to be less impulsive than their male counterparts (e.g., Perez et al., 2016) and this trait has been found to peak in adolescence and show a decline afterward until adulthood (e.g., Inuggi et al., 2014; Steinberg et al., 2008); indeed, over time, adolescents develop cognitive and emotional skills typical of adulthood (e.g., Steinberg, 2008), leading to a stabilization in the frequency of impulsive behavior in the early to mid-20s (Steinberg et al., 2008). Moreover, regarding the family structure and the parent characteristics subsystems, higher parental socioeconomic status has been found to predict adolescents’ lower impulsivity (Assari et al., 2018) and higher maternal self-control to predict adolescents’ higher self-control directly and indirectly via sequential associations with maternal attachment and effective parenting (Bolger et al., 2022). This last finding is in line with one previous study that found that lower parental impulsivity is associated with adolescent’s higher self-control (Turiuc & Pojoga, 2018).

Regarding the peers and the community subsystems, adolescents and young adults higher in impulsivity have been found to report fewer stable relationships with others (Zhang & Lin, 2015), with this trait being negatively associated with peer trust (Moyano et al., 2022) and school connectedness (Joo & Lee, 2020). Indeed, a positive school environment and positive peer relationships established in this context contribute to a more adaptive response to stressful situations, through the development of higher self-control (Joo & Lee, 2020). Specifically regarding aspects related to the family processes subsystem, impulsivity has been negatively, yet weakly, linked with early memories of warmth and safeness (Barreto Carvalho et al., 2015), positive parenting (Cassels et al., 2022), closeness with family (Song et al., 2019), as well as trust and communication with parents (Moyano et al., 2022). Indeed, aspects of positive parenting (e.g., positive early child-parent interactions) have been found to play an essential role in the development of impulse control in youth (e.g., Khurana & Romer, 2020; Scott et al., 2009), indicating a causal association between positive parenting and this trait; on the other hand, impulsive children may also be more difficult to parent (Johnston & Mash, 2001) and suggesting that impulsivity in youth may be an antecedent of negative parenting. However, one recent study (Cassels et al., 2022) did not find a causal relationship between these variables.

With regard to psychological traits, impulsivity has negatively associated with rational decision-making style (Jelihovschi et al., 2018), resilience (Ran et al., 2022), problem-focused coping (Li et al., 2019), and emotion regulation (e.g., Hasking & Claes, 2020). Indeed, a more rational cognitive style is characterized by a well-thought and logical evaluation of all possible options in a decision-making situation (Scott & Bruce, 1995) and generally involves a reasonable and balanced examination of the positive and negative consequences of each decision, contrary to the lack of planning involved in impulsivity. Additionally, the negative associations between resilience, coping, emotion dysregulation, and impulsivity may be explained by the fact that high self-control opposite to this trait is a relevant factor linked to the enhancement of resilience (e.g., Wills & Bantum, 2012), with people higher in self-control being more resilient to adverse experiences given they are more likely to use adaptive emotion regulation strategies (e.g., cognitive reappraisal) to reduce the perceived threat of a problematic situation (Mischel et al., 2011). This is also in line with the finding that young people with higher emotion regulation are less likely to engage in impulsive behaviors to regulate their negative emotions and to show difficulties in anticipating the consequences of their actions (Hasking & Claes, 2020).

## Current Study

Multiple previous studies have examined the negative associations between many psychosocial factors and impulsivity in adolescence (e.g., gender, age, positive family characteristics, positive school context, positive peer relationships, rational decision-making style, emotion regulation), either as antecedents of this trait—therefore having a causal relationship with it—or as negative correlates. However, there is a paucity of research examining the contribution of multiple negative correlates/protective factors for adolescent impulsivity nor exploring the moderating role of these variables in the (positive) associations between this trait and risk-related factors. To address this gap, drawing on multiple components of the unified theory of development—including biological factors (i.e., gender, age), the peers subsystem (i.e., social group attachment, satisfaction with classmates, peers form other classes, and friends from school), the community subsystem (i.e., school satisfaction, satisfaction with teachers, and school staff), the family processes subsystem (i.e., early memories of warmth and safeness, parental attachment, satisfaction with parents, siblings, and remaining family), and psychological variables (i.e., rational decision-making style, resilience, coping, emotion regulation) – this research explored to what extent multiple psychosocial variables are negatively related with impulsivity, both in younger (i.e., 13–15) and older (i.e., 16–19) adolescents and examined the moderating role of these variables in the (positive) relationships between this trait and risk-related outcomes. The hypotheses were based on the literature outlined previously. This study initially aimed to characterize the levels of impulsivity using a representative sample of adolescents living on all nine islands of the Azores archipelago (Portugal) by gender and specific age group (i.e., 13, 14, 15, 16, 17, and 18 or older). It was hypothesized that impulsivity would be higher in males and in younger adolescent age groups (i.e., 13, 14, 15, 16) (Hypothesis 1). The second aim was to examine the associations between impulsivity and some risk-related variables: verbal aggression, anger, self-harm, and other high-risk behaviors. It was hypothesized that impulsivity would positively associate with all these risk-related outcomes (Hypothesis 2). As stated earlier, the primary aim of the study was to explore to what degree multiple psychosocial variables are negatively related with this trait that characterizes typical adolescent development, both in younger (i.e., 13–15) and older (i.e., 16–19) adolescents. It was hypothesized that impulsivity would be negatively linked, in both age groups, with an array of psychosocial variables (Hypothesis 3a) that would, then, explain a significant portion of the variance in this trait in adolescence: early memories of warmth and safeness, rational decision-making style, resilience, emotion regulation, coping, parental attachment, social group attachment, satisfaction with school-related variables, and satisfaction with family-related variables (Hypothesis 3b). Lastly, this study aimed to examine the moderating role of the negative correlates/protective factors of impulsivity identified in this study in the associations between this trait and the risk-related variables mentioned above. It was hypothesized that all the negative correlates/protective factors of impulsivity would have a negative effect in the (positive) relationships between impulsivity and each risk-related variable (i.e., decrease the strength of these associations) (Hypothesis 4).

## Method

### Participants

This study is part of a research project entitled Vida+—described below (in the “Procedure and Ethics” section). The initial sample comprised a total of 8,622 adolescents, which corresponds to a vast majority of all students enrolled in the Portuguese public education system (*ensino regular público*) living on all the nine islands of the Autonomous Region of the Azores (Portugal), of which 704 were removed for either not having reported their age, being younger than 13, or being older than 19—considering the small sample size (*n* = 249) and in accordance with the adolescent age range defined by the WHO (n.d.)—and/or for not having stated their school year or for being in fourth or fifth grade—given the small number of participants in these school years (*n* = 2). Additionally, having ever received a psychological/psychiatric diagnosis was an exclusion criterion considering some mental health problems (e.g., depression, anxiety) involve and/or have been found to be strongly associated with impulsivity (e.g., Moustafa et al., 2017), with the focus of this study being the aspects of impulsivity related to a healthy development. Out of the total number of participants after excluding those due to age and school year (*n* = 7,918), 6,894 mentioned they had never received a psychiatric diagnosis, so this was the final sample size. Participants’ ages ranged from 13 to 19 (*M* = 15.4, *SD* = 1.7), with 3,248 (47.1%) identifying with the male gender and 3,644 (52.9%) with the female gender. At time of participation, most adolescents were in ninth grade (24.8%), seventh grade (22%), or eighth grade (21%), and had never failed a school year (65.1%). These sample characteristics are presented in Table 1. No data were collected about adolescents’ racial/ethnic characteristics nor socioeconomic status.

**Table 1:** Participants’ sociodemographic characteristics (*N* = 6894)

Sociodemographic characteristics	Sample	
	*n*	*%*
Gender		
Male	3248	47.1
Female	3644	52.9
Age groups		
13 years	1051	15.2
14 years	1231	17.9
15 years	1494	21.7
16 years	1230	17.8
17 years	924	13.4
≥18 years	964	14
School year^a^		
6th grade (11 years old)	46	0.7
7th grade (12 years old)	1517	22
8th grade (13 years old)	1451	21
9th grade (14 years old)	1708	24.8
10th grade (15 years old)	1000	14.5
11th grade (16 years old)	680	9.9
12th grade (17 years old)	492	7.1
Ever failed a school year		
Yes	2406	34.9
No	4485	65.1

^a^The ages most commonly associated with each Portuguese school year are presented in parentheses

### Measures

Multiple questionnaires and self-report measures of variables considered to decrease the risk for adolescent impulsivity, further described below, were included in the research protocol designed specifically for Vida+, the research project this study is part of—see the “Procedure and Ethics” section for more information on this project. In order to exclude the portion of participants who had received a psychological/psychiatric diagnosis (or who did not provide this information), the protocol included a question directly asking participants for this information.

#### Impulsivity

Impulsivity was assessed using the Impulse, Self-harm and Suicide Ideation Questionnaire for Adolescents (ISSIQ-A; Barreto Carvalho et al., 2015), a measure composed of 56 items scored on a Likert scale ranging from 0 = *Never happens* to 3 = *Always happens to me* and split into four sections represented by letters A to D, each measuring a different construct: impulsivity (section A; eight items; e.g., “Sometimes I have difficulty stopping a behavior even if it may hurt me”), self-harm (section B; eight items; e.g., “I scratch or pinch some parts of my body on purpose”) and other high-risk behaviors (section B; six items; e.g., “I use alcohol excessively”), automatic reinforcement (section C; 24 items; e.g., “I hurt myself to be able to feel something”) and social reinforcement (section C; seven items; e.g., “Hurting myself helps others understand my problems”) functions of self-harm, and suicidal ideation (section D; three items; e.g., “There have been times during which I thought I wanted to die”). A total score of impulsivity was computed using the corresponding items, with higher scores indicating greater impulsivity. In the original study (Barreto Carvalho et al., 2015), the impulsivity subscale showed acceptable internal consistency, α = 0.77. In this study, this subscale exhibited good internal consistency, α = 0.86. A confirmatory factor analysis using a one-factor solution was conducted in this subscale, with the RMSEA (0.11) value indicating poor model fit but the CFI (0.91), the NFI (0.90), and the TLI (0.90) values suggesting a good fit (Bentler, 1990; Fabrigar et al., 1999).

#### Possible Negative Correlates and Protective Factors of Impulsivity

Multiple psychosocial variables hypothesized to negatively associate with impulsivity were explored: early memories of warmth and safeness using the Early Memories of Warmth and Safeness for Adolescents Scale (EMWSS-A; original version by Richter et al., 2009; Portuguese version by Cunha et al., 2014), rational decision-making style using the General Decision-Making Style Scale (GDMSS; original version by Scott & Bruce, 1995; Brazilian version by Löbler et al., 2019), resilience using the Resilience Scale (RS; original version by Wagnild & Young, 1993; Portuguese version by Felgueiras et al., 2010), emotion regulation using the Situational Test of Emotional Management-Brief (STEM-B; original version by Allen et al., 2015; Portuguese version by da Motta et al., 2021), coping using the Toulousiana Coping Scale (TCS; original version by Esparbès et al., 1993; Portuguese adolescent version by Amaral-Bastos et al., 2015), parental attachment using the Inventory of Parent and Peer Attachment (IPPA; original version by Armsden & Greenberg, 1987; Portuguese version by Machado & Oliveira, 2007), social group attachment using the Social Group Attachment Scale (SGAS; original version by Smith et al., 1999; Portuguese version by Dinis et al., 2008), satisfaction with school-related variables using six self-report items (i.e., school satisfaction, satisfaction with classmates, peers from other classes, friends from school, teachers, school staff), and family satisfaction using three self-report items (i.e., satisfaction with parents, siblings, and remaining family). See Supplementary Text 1 for a more detailed description of these measures.

#### Aggression, Self-Harm, and Other High-Risk Behaviors

To examine verbal aggression, anger, self-harm, and other high-risk behaviors (e.g., substance use, reckless driving, risky sexual behavior), adolescents completed the Buss-Perry Aggression Questionnaire (AQ; original version by Buss & Perry, 1992; Portuguese version by Simões, 1993) and the ISSIQ-A (Barreto Carvalho et al., 2015; described above). See Supplementary Text 1 for a more detailed description of the AQ and for the internal consistencies of the ISSIQ-A self-harm and other high-risk behaviors subscales.

### Procedure and Ethics

This research is part of a project entitled Vida+ aiming to explore specific sociocultural and individual (e.g., disruptive emotional experiences, coping strategies, emotion regulation) variables influencing substance use in Azores, Portugal. This study was developed by the University of the Azores and received approval from the Ethics Committee of the University of the Azores and the Portuguese Data Protection Authority (no. 13953/2017). Multiple self-report measures and questionnaires were used in a research protocol developed specifically for Vida+. To maximize student participation, this protocol was administered both using paper and pencil format and digitally (e.g., via an electronic link) to students across Azorean schools on all islands of this Autonomous Region. Two different moments of participation were used to prevent effects of fatigue on students and maximize response accuracy.

All international ethical norms and standards, including anonymity and confidentiality of data, regarding research involving human participants (e.g., Declaration of Helsinki) were respected. An informed consent form was signed by all the underage participants’ parents/legal guardians and all participants above the age of 18.

#### Analytical strategy

Data were analyzed using SPSS version 27. As mentioned previously, participants (*n* = 1024) who reported they had received a psychological/psychiatric diagnosis and those who did not provide this information were excluded. Firstly, descriptive statistics (e.g., means, standard deviations) were computed for impulsivity by gender and age groups (Hypothesis 1). Gender was coded as a dichotomous variable with 0 = *Male* and 1 = *Female*. Secondly, Pearson correlations were conducted to examine the relationships between impulsivity and some risk-related variables (i.e., verbal aggression, anger, self-harm, other high-risk behaviors) (Hypothesis 2). Further correlations were computed to examine the associations between impulsivity and the variables hypothesized to negatively associate with this trait (i.e., early memories of warmth and safeness, rational decision-making style, resilience, emotion regulation, coping, parental attachment, social group attachment, satisfaction with school-related variables, family satisfaction) both in the younger (i.e., 13–15) and the older (i.e., 16–19) adolescents (Hypothesis 3a); afterward, two multiple linear regression models—one for each age group—using the stepwise method were conducted using all variables that significantly (negatively) correlated with impulsivity as the explanatory (i.e., independent) variables and impulsivity as the outcome (i.e., dependent) variable (Hypothesis 3b). Lastly, to examine the moderating role of the negative correlates/protective factors of impulsivity in the associations between this trait and each risk-related variable, moderation analyses were conducted in the PROCESS macro using SPSS (Hayes, 2013) (Hypothesis 4). Correlation coefficients lower than |0.20| were considered weak, those between |0.20| and |0.50| moderate, and those greater than |0.50| strong (Ferguson, 2009). The level of significance used for all analyses was *p* < 0.05. In all analyses, missing data were excluded pairwise (i.e., correlations, *t*-tests, ANOVAs, chi-square tests) or listwise (i.e., stepwise regressions, moderations) considering the large sample size.

## Results

### Characterization of Impulsivity

The means and standard deviations for the impulsivity measure are presented in Table 2. A mean of 7.9 (SD = 4.8) of impulsivity was shown by the total sample, which indicates that participating adolescents tended to exhibit low mean levels of this variable. Male adolescents (*M* = 8.1, SD = 5.1) showed significantly higher levels of impulsivity than females (*M* = 7.8, SD = 4.4), *t*(5632.06) = 2.39, *p* = 0.017. Impulsivity was significantly different across age groups, *F*(5, 6163) = 3.08, *p* = 0.009, with 13-year-old adolescents showing lower levels than 14 and 16-year-olds. These results only provide partial support for Hypothesis 1 – that males and younger adolescents (i.e., 13, 14, 15, 16) would be higher in impulsivity.

**Table 2:** Descriptives of Impulsivity by Gender and Age Group

		Gender		Age group					
	Total sample	Males	Females	13 years	14 years	15 years	16 years	17 years	≥18 years
	*M* (SD)	*M* (SD)	*M* (*SD*)	*M* (SD)	*M* (SD)	*M* (SD)	*M* (SD)	*M* (SD)	*M* (SD)
Impulsivity	7.9 (4.8)	8.1 (5.1)	7.8 (4.4)	7.5 (4.8)	8.2 (4.8)	7.7 (4.8)	8.1 (4.8)	8.1 (4.6)	7.9 (4.7)
*t*		2.39							
*F*				3.08					
*p*		<0.001		0.012					
*Cohen’s d*		0.06							
*η*^2^				0.01					

### Associations between Impulsivity and Risk-related Variables

To examine the relationships between impulsivity and some risk-related variables (i.e., verbal aggression, anger, self-harm, other high-risk behaviors), multiple Pearson correlations were conducted. Impulsivity was positively associated (*p* < 0.001) with all of the variables examined, *r*s ranging from 0.33 for verbal aggression to 0.37 for self-harm. Therefore, despite the adolescent community sample used in this study exhibiting low mean levels of impulsivity, these results suggest that this trait still plays a relevant role in the engagement in high-risk behaviors (similar to at-risk samples), so it is relevant to examine possible psychosocial factors that negatively associate with impulsivity.

### Correlations

Correlation analyses were conducted between impulsivity and the variables hypothesized to negatively associate with this trait in both younger (i.e., 13–15) and older (i.e., 16–19) adolescents. All coefficients are presented in Appendices A (younger adolescents) and B (older adolescents). In both age groups, non-statistically significant associations were found between impulsivity and gender, age, satisfaction with peers from other classes, and satisfaction with friends from school. Only in older adolescents, impulsivity was not associated with resilience, satisfaction with classmates, peers from other classes, school staff, and with siblings. Furthermore, in both age groups, impulsivity was positively associated (*p* < 0.001) with coping, *r* = 0.14 in younger adolescents and 0.09 in their older counterparts. Lastly, in both age groups, negative correlations were found for early memories of warmth and safeness, rational decision-making style, emotion regulation, parental attachment, social group attachment, school satisfaction, satisfaction with teachers, parents, and remaining family, with *r*s ranging from −0.05 (*p* = 0.002) for satisfaction with classmates in younger adolescents and −0.04 (*p* = 0.048) for satisfaction with remaining family in older adolescents to −0.27 and −0.22 (*p* < 0.001) for parental attachment in younger and older adolescents, respectively. Only in younger adolescents, impulsivity was negatively associated with resilience, satisfaction with classmates, school staff, and siblings, with *rs* ranging from −0.05 (*p* = 0.003) for resilience and satisfaction with classmates and −0.09 (*p* < 0.001) for satisfaction with school staff.

The variables that were negatively associated with impulsivity (i.e., early memories of warmth and safeness, rational decision-making style, resilience, emotion regulation, parental attachment, social group attachment, school satisfaction, satisfaction with classmates, teachers, school staff, parents, siblings, and remaining family) were included as explanatory (i.e., independent) variables in the two multiple linear regression models (one for each age group) using impulsivity as the outcome (i.e., dependent) variable presented below.

### Multiple Linear Regression

Preliminary analyses indicated that all assumptions for multiple regression were met in both younger (i.e., 13–15) and older (i.e., 16–19) adolescents, namely: linearity, normality of residuals (i.e., bell-shaped histograms), non-multicollinearity (i.e., all correlations below 0.90, all VIF values below 10, and all tolerance value greater than 0.2), homoscedasticity (i.e., scatterplots of standardized residuals show a random array of dots around zero), independence of errors (i.e., both Durbin-Watson values between one and three). Two multiple linear regressions—one for each age group—using the stepwise method using impulsivity as the outcome variable were conducted. At each step, explanatory variables were chosen based on *p* values (*p* ≤ 0.05 for entry and ≥0.10 for removal). The results of these regressions are presented in Appendices C (younger adolescents) and D (older adolescents).

Starting with the 13 variables that negatively associated with impulsivity in the younger adolescents (i.e., early memories of warmth and safeness, rational decision-making style, resilience, emotion regulation, parental attachment, social group attachment, school satisfaction, satisfaction with classmates, teachers, school staff, parents, siblings, and remaining family), the stepwise regression reduced them to the following four in the final (fourth) model, by decreasing regression coefficients: parental attachment, *β* = −0.17, *p* < 0.001, social group attachment, *β* = −0.14, *p* < 0.001, satisfaction with teachers, *β* = −0.12, *p* < 0.001, and emotion regulation, *β* = −0.07, *p* = 0.006. This model was statistically significant, *F*(4, 1429) = 41.92, *p* < 0.001, and explained 10.3% of the variance in impulsivity (based on the adj. *R*^2^). Additionally, starting with the nine variables that negatively associated with impulsivity in the older adolescents (i.e., early memories of warmth and safeness, rational decision-making style, emotion regulation, parental attachment, social group attachment, school satisfaction, satisfaction with teachers, parents, and remaining family), the stepwise regression reduced them to the following three in the final (third) model, by decreasing regression coefficients: emotion regulation, *β* = −0.17, *p* < 0.001, parental attachment, *β* = −0.15, *p* < 0.001, and social group attachment, *β* = −0.14, *p* < 0.001. This model was statistically significant, *F*(3, 1711) = 65.67, *p* < 0.001, and explained 10.2% of the variance in impulsivity (based on the adj. *R*^2^).

In sum, emotion regulation, parental attachment, and social group attachment had a negative effect on adolescent impulsivity in both younger (i.e., 13–15) and older (i.e., 16–19) age groups, with satisfaction with teachers having had this effect on impulsivity only in younger adolescents. These findings only provide partial support to Hypothesis 2—that early memories of warmth and safeness, rational decision-making style, resilience, emotion regulation, parental and social group attachment, satisfaction with school-related variables, and satisfaction with family related-variables would have a negative effect on impulsivity in both age groups.

### Moderations

To examine the moderating role of each variable that negatively associated with impulsivity in at least one age group (i.e., emotion regulation, parental attachment, social group attachment, satisfaction with teachers) in the relationships between impulsivity and the risk-related variables mentioned above (i.e., verbal aggression, anger, self-harm, other high-risk behaviors), a total of 16 moderated regressions using the PROCESS macro in SPSS (Hayes, 2013) was conducted in the total sample. The results of these regressions are presented in Appendix E. Significant interaction effects between impulsivity and emotion regulation, as well as between impulsivity and parental attachment, were found in the models using self-harm and other high-risk behaviors as the outcome variables; significant interaction effects between impulsivity and social group attachment were found in all models; lastly, no significant interaction effects between impulsivity and satisfaction with teachers were found in any models. In all models where a significant interaction effect was found, the negative correlates/protective factors of impulsivity were negative moderators in the associations between impulsivity and each risk-related variable; in other words, the former variables weakened the positive effect of impulsivity on each risk-related variable. See Supplementary Text 2 for a detailed description of the simple slopes analyses. Additionally, given that satisfaction with teachers only emerged as a factor that negatively associated with impulsivity in the younger adolescents (i.e., 13–15), four further moderated regressions were conducted to examine the moderating role of this variable in the relationships between impulsivity and each risk-related variable in this subsample. Similar to what was found in the overall sample, no significant interaction effect between impulsivity and satisfaction with teachers was found on each risk-related variable.

### Sensitivity Analyses

Given that adolescent age was dichotomized relatively arbitrarily (i.e., younger adolescents were considered those who are 13–15 years old and older adolescents were considered those who are 16–19 years old), sensitivity analyses were conducted to explore whether the negative correlates/protective factors of impulsivity differed across the specific age groups (i.e., 13, 14, 15, 16, 17, 18, or older). Further analyses were conducted to examine whether these variables differed by gender (i.e., four gender groups: females aged 13–15, females 16–19, males 13–15, males 16–19). See Supplementary Text 3 and Supplementary Tables for the results and their descriptions. The results differed to a certain extent across groups: similar to the findings, parental attachment and social group attachment had a negative effect on impulsivity across younger and older age groups (both had this effect in the adolescents who are 13, 14, 15, and 16; the former had this effect across all age groups except for those who are and 18 or older; the latter had this effect across all ages except for the 17-year-olds) and in all gender groups, with satisfaction with teachers only emerging as a protective factor in the 14-year-olds and in the younger females, and rational decision-making style in those who are 18 or older and in the older females; lastly, emotion regulation had a negative effect on impulsivity in all older age groups (i.e., 16, 17, 18, or older), in the 14-year-olds, and in all gender groups except for the younger females.

## Discussion

The literature shows that impulsivity, a particularly prevalent characteristic in adolescence, is associated with an increased risk for multiple negative outcomes, with previous studies having found that it is negatively linked with many psychosocial variables, either as being its antecedents or as negative correlates. However, there is limited research exploring to what degree multiple negative correlates/protective factors for adolescent impulsivity nor examining the moderating role of these variables in the (positive) relationships between this trait and maladaptive outcomes. To address this gap, drawing on multiple aspects of the unified theory of development (Sameroff, 2010)—namely biological and psychological factors, as well as variables related to the social context (i.e., peers, school, family)—and using a representative sample of 6,894 adolescents living on all nine islands of the Azorean archipelago (Portugal), this study examined to what extent an array of psychosocial variables is negatively associated with impulsivity, both in younger (i.e., 13–15) and older (i.e., 16–19) adolescents, and explored the moderating role of those that have negative associations with this trait in the relationships between impulsivity and some risk-related variables (i.e., verbal aggression, anger, self-harm, other high-risk behaviors). In both age groups, the psychological system and all subsystems of the social context explored (i.e., peers, community, family processes) played a role in the explanation of impulsivity, with only emotion regulation, parental attachment, and social group attachment having had a negative effect on this trait; furthermore, these variables weakened many (positive) associations between impulsivity and each risk-related variable.

This research initially aimed to characterize impulsivity by gender and specific age group (i.e., 13, 14, 15, 16, 17, and 18, or older). Low mean levels of impulsivity were found in the sample, with male adolescents showing higher levels of this trait than females, which is in line with previous research (e.g., Perez et al., 2016). Impulsivity was found to be higher in the 14 and 16-year-old adolescents compared to the 13-year-olds, which does not support the initial hypothesis based on research that shows that impulsivity tends to peak in adolescence and decline between late adolescence and early adulthood (e.g., Inuggi et al., 2014; Steinberg et al., 2008). The finding that 13-year-olds exhibited lower impulsivity is likely due to the higher parental supervision that these individuals are subjected to (compared to those who are older), which may serve as an inhibitory mechanism for impulsivity in early adolescence. These findings suggest that it is particularly more relevant to examine adolescent impulsivity and related predictors and outcomes starting at the beginning of middle adolescence (i.e., 14 years). Consistent with previous research, impulsivity was positively associated with an array of risk-related variables, namely verbal aggression and anger (e.g., Cao & An, 2020), self-harm (e.g., Hasking & Claes, 2020), and other high-risk behaviors (e.g., substance use, reckless driving, risky sexual behavior) (e.g., Hentges et al., 2018; VanderVeen et al., 2016), which is evidence for the idea that impulsivity in this sample has similar characteristics to that of other adolescent samples in previous studies examining this trait. Moreover, these positive associations reveal that despite being a community sample, higher impulsivity is associated with higher endorsement of risk behaviors, increasing the relevance of studying the factors that negatively associate with this trait in this sample.

In both younger (i.e., 13–15) and older (i.e., 16–19) adolescents, impulsivity was positively associated with coping in both age groups, not in line with a previous study (Li et al., 2019) that found that this trait was negatively associated with problem-focused coping, which suggests that adolescents who have better coping skills are also more likely to engage in risk-taking because they will be more able to deal with problematic situations that may arise from impulsive behaviors. On the other hand, in both age groups, impulsivity was negatively linked with many variables also previously associated with this trait: early memories of warmth and safeness (Barreto Carvalho et al., 2015); rational decision-making style (Jelihovschi et al., 2018); emotion regulation (e.g., Hasking & Claes, 2020); parental attachment, and some satisfaction with family-related variables (i.e., parents, remaining family) – which is in line with previous findings that impulsivity is negatively linked with positive parenting (Cassels et al., 2022), closeness with family (Song et al., 2019), and trust and communication with parents (Moyano et al., 2022); social group attachment (Moyano et al., 2022); and some satisfaction with school-related variables (i.e., school, teachers) (Joo and Lee, 2020). Only in younger adolescents, impulsivity was negatively associated with resilience and some satisfaction with family (i.e., siblings) and school-related (i.e., classmates, school staff) variables.

Both regression models (for younger and older adolescents) explained relatively small variances in impulsivity (i.e., ~10% in both age groups), which indicates that a larger portion of impulsivity across the adolescent period is better explained (as initially expected) by other aspects unrelated to these psychosocial factors (e.g., biological), in line with the fact that this trait has a strong neurobiological component and is adaptive from an evolutionary perspective (e.g., Steinberg, 2008). In both younger (i.e., 13–15) and older (i.e., 16–19) adolescents, only emotion regulation, parental attachment, and social group attachment had a negative effect on impulsivity. In addition to the contextual model (mentioned above), these findings may be explained in light of the regulation model within the unified theory of development (Sameroff, 2010), according to which self-regulation is developed through social interactions, firstly with primary caregivers (e.g., parents) and then other significant figures (e.g., peers), and progresses from biological (e.g., hunger) to more psychological and social processes such as impulse control. Therefore, emotion regulation should be a target for intervention when trying to reduce impulsivity given its benefits in the prevention and/or mitigation of risks and vulnerabilities in adolescence including substance use (Barahmand et al., 2016), risky sexual behaviors (Hessler & Katz, 2010), self-harm (e.g., Barreto Carvalho et al., 2023a, 2023b), and suicidal ideation (e.g., Quintana-Orts et al., 2020). Additionally, the findings emphasize the negative association between adolescent impulsivity and positive social relationships across the full developmental period, in line with the previous finding that less impulsive young people are more prone to show more sable relationships (Zhang & Lin, 2015). Particularly, the negative relationship between parental attachment and impulsivity suggests that positive parenting skills that promote a secure attachment (e.g., adequate parental supervision)—which can and should be a target of intervention—improve impulse control and increase exploratory (i.e., adaptive) risk-taking, ultimately contributing to a healthy adolescent development. Furthermore, the negative association between social group attachment and impulsivity is aligned with previous research showing that peer trust is negatively linked with impulsivity (Moyano et al., 2022). Indeed, previous research shows that peer influence plays an essential role in adolescent risk-taking (Chein et al., 2011); it is possible that adolescents high in social group attachment will likely be more able to express themselves within their group and other members will also be more open to provide support, and this will likely have a positive impact on group decision-making, leading to fewer impulsive actions. Only in younger adolescents, satisfaction with teachers negatively associated with impulsivity, therefore it should be enhanced by promoting healthier relationships between these professionals and adolescents—for example, using interventions/programs targeting socioemotional skills (e.g., assertiveness, conflict resolution)—as well as healthier school environments more broadly, and more indirectly, motivation for learning and school engagement more generally.

Even though impulsivity has a strong neurobiological component (Steinberg, 2008), the variables that negatively associated with impulsivity in both age groups (i.e., emotion regulation, parental attachment, social group attachment) have a much stronger environmental/social component, revealing that the psychological system and all subsystems of the social context measured (i.e., peers, community, family processes) play a relevant role in adolescent impulsivity. All these results suggest that promoting emotion regulation, positive parenting practices, and healthier relationships with teachers will allow for a more adaptive experimentation and exploration, typical of adolescent impulsivity (Icenogle & Cauffman, 2021; Steinberg, 2008).

Lastly, the interaction effects between impulsivity and its negative correlates/protective factors (i.e., emotion regulation, parental attachment, social group attachment) on a variety of risk-related variables (i.e., verbal aggression, anger, self-harm, other high-risk behaviors) were examined and emotion regulation and parental attachment weakened the positive associations between impulsivity and self-harm and other high -risk behaviors (e.g., substance use, reckless driving, risky sexual behavior); additionally, social group attachment weakened the positive relationships between this trait and all risk-related variables (i.e., verbal aggression, anger, self-harm, other high-risk behaviors); lastly, satisfaction with teachers did not show this effect in the associations between impulsivity and any risk-related variables. This last result could have been explained by the fact that satisfaction with teachers only had a negative effect on impulsivity in the younger adolescents, so a further moderation was conducted in this subsample; however, in the younger individuals, there was also no moderation effect of this variable in the associations between this trait and the risk-related variables. This may be explained by the fact that throughout adolescence, it is very likely that youth have an array of teachers, with many not teaching the same classes across multiple school years, consistently, making it difficult for adolescents to establish strong and stable positive relationships with these educators and making them less likely to dampen the positive effect of impulsivity on multiple risk-related outcomes (compared to the other negative correlates/protective factors of impulsivity). Regardless, these results provide evidence for the notion that emotion regulation, parental attachment, and social group attachment have an interaction effect with impulsivity that decreases its positive effect on the other risk-related variables (i.e., verbal aggression, anger, self-harm, other high-risk behaviors); in other words, these results suggest that these factors have a relevant role in the reduction of the risk that impulsivity has on the endorsement of multiple risk-related outcomes.

Some limitations of the study must be considered, including its cross-sectional nature, which makes it impossible to accurately infer predictive relationships between the explanatory variables and impulsivity over time; the use of self-report measures, including some retrospective scales (e.g., EMWS-A), which may lead to a social desirability bias (Krumpal, 2013); and the use of a lengthy research protocol, which may have had a negative impact on adolescents’ energy and motivation, even though two different moments for participation were used. Furthermore, another limitation of the study is not having been able to assess and statistically control for other variables—particularly those related to the subsystems of the social context that were not explored (e.g., family structure, parent characteristics)—that have an effect on adolescent impulsivity and might interact with some independent variables to influence this trait, such as the levels of parental socioeconomic status (Assari et al., 2018) and parental impulsivity (Bolger et al., 2022; Turiuc & Pojoga, 2018). Lastly, one limitation is the fact that impulsivity was measured as a unidimensional construct using a subscale of a measure specifically developed to assess adolescent self-harm (Barreto Carvalho et al., 2015), despite this subscale being comprised of eight items. Future studies should use specific measures of impulsivity to examine whether these findings are replicated, and other explanatory variables may also be explored, including other aspects related to early relationships (e.g., parental styles and practices), social relationships (e.g., association with prosocial/anti-social peers), and to the school context (e.g., student engagement, school achievement). Lastly, future research should use longitudinal designs to determine the consistency (or lack thereof) of the negative correlates/protective factors of adolescent impulsivity or identify whether they differ across different phases of adolescence (e.g., early, middle, late). The findings of this research will allow professionals who work with adolescents to promote psychosocial factors that enhance the positive aspects of the naturally high impulsivity in adolescence across multiple stages.

## Conclusion

Impulsivity is often high in adolescence, with some studies showing that this trait is negatively associated with multiple psychosocial factors. Drawing on multiple aspects of the unified theory of development—namely biological and psychological factors, as well as variables related to the social context (i.e., peers, school, family)—using a representative sample of adolescents living on all nine islands of the Azores archipelago, this study aimed to identify to what extent multiple psychosocial variables are negatively associated with impulsivity, in both younger (i.e., 13–15) and older (i.e., 16–19) adolescents, as well as to explore the moderating role of the negative correlates/protective factors of this trait in the associations between impulsivity and some risk-related variables. In both age groups, emotion regulation, parental attachment, and social group attachment had a negative effect on impulsivity, with satisfaction with teachers only having had this effect in the younger adolescents; moreover, most of these negative correlates/protective factors of impulsivity weakened the positive associations between this trait and some of the risk-related variables. These findings indicate that the psychological system and all subsystems of the social context examined influence adolescent impulsivity and that promoting emotion regulation, positive parenting practices, healthier relationships with peers (in both age groups), and healthier relationships with teachers (particularly in younger adolescents) likely reduces this trait in this developmental period; targeting these aspects related to impulse-control will allow for a more adaptive experimentation and exploration typical of adolescent impulsivity. On the other hand, the relatively small variances in impulsivity explained suggest that this trait is better explained by other aspects unrelated to these psychosocial factors (e.g., biological).

### Supplementary Information

Supplementary Material

## Appendix

Table 3, Table 4, Table 5, Table 6, Table 7

**Table 3:** Pearson correlation coefficients between impulsivity and possible negative correlates and protective factors in younger adolescents (13–15)

	Impulsivity	
	*n*	*r*
Gender	3337	−0.03
Age	3337	0.01
Early memories of warmth and safeness	3237	−0.14^***^
Rational decision-making style	2365	−0.07^***^
Resilience	2955	−0.05^**^
Emotion regulation	3334	−0.16^***^
Coping	2400	0.14^***^
Parental attachment	2401	−0.27^***^
Social group attachment	2090	−0.24^***^
School satisfaction	3328	−0.12^***^
Satisfaction with classmates	3327	−0.05^**^
Satisfaction with peers from other classes	3329	−0.01
Satisfaction with friends from school	3325	−0.02
Satisfaction with teachers	3336	−0.15^***^
Satisfaction with school staff	3334	−0.09^***^
Satisfaction with parents	3327	−0.07^***^
Satisfaction with siblings	3081	−0.07^***^
Satisfaction with remaining family	3103	−0.06^**^

Impulsivity was measured using the Impulse, Self-harm and Suicide Ideation Questionnaire for Adolescents (ISSIQ-A; Barreto Carvalho et al., 2015). Rational decision-making style was measured using the General Decision-Making Style Scale (GDMSS; Scott & Bruce, 1995; Brazilian version by Löbler et al., 2019). Resilience was measured using the Resilience Scale (RS; Wagnild & Young, 1993; Portuguese version by Felgueiras et al., 2010). Emotion regulation was measured using the Situational Test of Emotion Management-Brief (STEM-B; Allen et al., 2015; Portuguese version by da Motta et al., 2021). Coping was measured using the Toulousiana Coping Scale (TCS; Esparbès et al., 1993; Portuguese version by Amaral-Bastos et al., 2015). Parental attachment was measured using the Inventory of Parent and Peer Attachment (IPPA; Armsden & Greenberg, 1987; Portuguese version by Machado & Oliveira, 2007). Social group attachment was measured using the Social Group Attachment Scale (SGAS; Smith et al., 1999; Portuguese version by Dinis et al., 2008).

^*^*p* < 0.05; ^**^*p* < 0.01; ^***^*p* < 0.001

**Table 4:** Pearson correlation coefficients between impulsivity and possible negative correlates and protective factors in older adolescents (16–19)

	Impulsivity	
	*n*	*r*
Gender	2836	−0.03
Age	2838	−0.02
Early memories of warmth and safeness	2761	−0.07^***^
Rational decision making-style	2079	−0.11^***^
Resilience	2510	0.04
Emotion regulation	2834	−0.21^***^
Coping	2092	0.09^***^
Parental attachment	2115	−0.22^***^
Social group attachment	1946	−0.20^***^
School satisfaction	2826	−0.05^**^
Satisfaction with classmates	2836	−0.03
Satisfaction with peers from other classes	2830	−0.02
Satisfaction with friends from school	2832	0.01
Satisfaction with teachers	2836	−0.08^***^
Satisfaction with school staff	2833	−0.03
Satisfaction with parents	2836	−0.06^**^
Satisfaction with siblings	2688	−0.03
Satisfaction with remaining family	2714	−0.04^*^

Impulsivity was measured using the Impulse, Self-harm and Suicide Ideation Questionnaire for Adolescents (ISSIQ-A; Barreto Carvalho et al., 2015). Rational decision-making style was measured using the General Decision-Making Style Scale (GDMSS; Scott & Bruce, 1995; Brazilian version by Löbler et al., 2019). Resilience was measured using the Resilience Scale (RS; Wagnild & Young, 1993; Portuguese version by Felgueiras et al., 2010). Emotion regulation was measured using the Situational Test of Emotion Management-Brief (STEM-B; Allen et al., 2015; Portuguese version by da Motta et al., 2021). Coping was measured using the Toulousiana Coping Scale (TCS; Esparbès et al., 1993; Portuguese version by Amaral-Bastos et al., 2015). Parental attachment was measured using the Inventory of Parent and Peer Attachment (IPPA; Armsden & Greenberg, 1987; Portuguese version by Machado & Oliveira, 2007). Social group attachment was measured using the Social Group Attachment Scale (SGAS; Smith et al., 1999; Portuguese version by Dinis et al., 2008).

^*^*p* < 0.05; ^**^*p* < 0.01; ^***^*p* < 0.001

**Table 5:** Results of the stepwise multiple linear regression of impulsivity in younger adolescents (13–15) (*N* = 3776)

	Model 1				Model 2				Model 3				Model 4			
	*B*	*SE*	*β*	*t*	*B*	*SE*	*β*	*t*	*B*	*SE*	*β*	*t*	*B*	*SE*	*β*	*t*
Independent variables																
Parental attachment	−1.67	0.17	−0.26	−10.08^***^	−1.34	0.17	−0.21	−7.71^***^	−1.18	0.18	−0.18	−6.70^***^	−1.08	0.18	−0.17	−6.04^***^
Social group attachment					−0.96	0.17	−0.15	−5.23^***^	−0.92	0.17	−0.14	−5.33^***^	−0.90	0.17	−0.14	−5.23^***^
Satisfaction with teachers									−0.71	0.15	−0.13	−4.89^***^	−0.65	0.15	−0.12	−4.42^***^
Emotion regulation													−0.11	0.04	−0.07	−2.76^**^
Model statistics																
* R*^2^	0.066				0.086				0.101				0.105			
Δ*R*^2^					0.020				0.015				0.005			
Adj. *R*^2^	0.066				0.084				0.099				0.103			
* F*	101.54^***^				66.73^***^				53.17^***^				41.92^***^			
Δ*F*					30.52^***^				23.88^***^				7.60^**^			

Parental attachment was measured using the Inventory of Parent and Peer Attachment (IPPA; Armsden & Greenberg, 1987; Portuguese version by Machado & Oliveira, 2007). Social group attachment was measured using the Social Group Attachment Scale (SGAS; Smith et al., 1999; Portuguese version by Dinis et al., 2008). Emotion regulation was measured using the Situational Test of Emotional Management-Brief (STEM-B; Allen et al., 2015; Portuguese version by da Motta et al., 2021). The unstandardized, as well as the standardized, regression coefficients are presented.

^*^*p* < 0.05; ^**^*p* < 0.01; ^***^*p* < 0.001

**Table 6:** Results of the stepwise multiple linear regression of impulsivity in older adolescents (16–19) (*N* = 3118)

	Model 1				Model 2				Model 3			
	*B*	*SE*	*β*	*t*	*B*	*SE*	*β*	*t*	*B*	*SE*	*β*	*t*
Independent variables												
Parental attachment	−1.51	0.15	−0.24	−9.99^***^	−1.22	0.15	−0.19	−7.97^***^	−0.95	0.16	−0.15	−6.02^***^
Emotion regulation					−0.26	0.04	−0.18	−7.51^***^	−0.25	0.04	−0.17	−7.30^***^
Social group attachment									−0.90	0.16	−0.14	−5.78^***^
Model statistics												
* R*^2^	0.055				0.086				0.103			
Δ*R*^2^					0.031				0.018			
Adj. *R*^2^	0.054				0.085				0.102			
* F*	99.69^***^				80.14^***^				65.67^***^			
Δ*F*					56.35^***^				33.46^***^			

Parental attachment was measured using the Inventory of Parent and Peer Attachment (IPPA; Armsden & Greenberg, 1987; Portuguese version by Machado & Oliveira, 2007). Social group attachment was measured using the Social Group Attachment Scale (SGAS; Smith et al., 1999; Portuguese version by Dinis et al., 2008). Emotion regulation was measured using the Situational Test of Emotional Management-Brief (STEM-B; Allen et al., 2015; Portuguese version by da Motta et al., 2021). The unstandardized, as well as the standardized, regression coefficients are presented.

^*^*p* < 0.05; ^**^*p* < 0.01; ^***^*p* < 0.001

**Table 7:** Moderated regressions of verbal aggression, anger, self-harm, and other high-risk behaviors on impulsivity, emotion regulation, parental attachment, social group attachment, and satisfaction with teachers

Regression models	*B*	95% CI		*t*	*p*	*F*	*p*	*R*^2^
		*LL*	*UL*					
DV: Verbal aggression						181.10	<0.001	0.109
Impulsivity	0.05	0.05	0.06	22.69	<0.001			
Emotion regulation (ER)	0.01	0.01	0.02	2.78	0.005			
Impulsivity × ER	0.01	−0.01	0.01	1.42	0.155			
Δ*R*^2^ = 0.001						2.02	0.155	
DV: Verbal aggression						176.39	<0.001	0.110
Impulsivity	0.05	0.05	0.06	21.12	<0.001			
Parental attachment (PA)	−0.01	−0.01	−0.01	−2.14	0.033			
Impulsivity × PA	−0.01	−0.01	0.01	−0.50	0.618			
Δ*R*^2^ = 0.001						0.25	0.618	
DV: Verbal aggression						224.07	<0.001	0.149
Impulsivity	0.04	0.04	0.05	17.51	<0.001			
Soc. group attach. (SGA)	−0.22	−0.25	−0.19	−13.72	<0.001			
Impulsivity ×SGA	−0.01	−0.02	−0.01	−3.40	0.001			
Δ*R*^2^ = 0.003						11.53	0.001	
DV: Verbal aggression						180.92	< 0.001	0.109
Impulsivity	0.05	0.05	0.06	22.48	<0.001			
Satisf. with teachers (ST)	−0.03	−0.06	−0.01	−2.49	0.013			
Impulsivity × ST	−0.01	−0.01	0.01	−0.23	0.816			
Δ*R*^2^ = 0.001						0.05	0.816	
DV: Verbal aggression^a^						92.28	<0.001	0.104
Impulsivity	0.05	0.04	0.05	15.77	<0.001			
Satisf. with teachers (ST)	−0.04	−0.08	−0.01	−2.37	0.018			
Impulsivity × ST	−0.01	−0.01	0.01	−0.37	0.712			
Δ*R*^2^ = 0.001						0.14	0.712	
DV: Anger						224.95	<0.001	0.132
Impulsivity	0.06	0.05	0.06	25.30	<0.001			
Emotion regulation (ER)	0.01	0.01	0.01	2.54	0.011			
Impulsivity × ER	0.01	−0.01	0.01	1.79	0.074			
Δ*R*^2^ = 0.001						3.20	0.074	
DV: Anger						246.35	<0.001	0.148
Impulsivity	0.05	0.05	0.05	22.50	<0.001			
Parental attachment (PA)	−0.01	−0.01	−0.01	−8.15	<0.001			
Impulsivity × PA	0.01	−0.01	0.01	−0.28	0.781			
Δ*R*^2^ = 0.001						0.08	0.781	
DV: Anger						348.51	<0.001	0.214
Impulsivity	0.04	0.04	0.05	19.36	<0.001			
Soc. group attach. (SGA)	−0.30	−0.33	−0.27	−20.27	<0.001			
Impulsivity × SGA	−0.01	−0.01	−0.01	−2.53	0.011			
Δ*R*^2^ = 0.001						6.40	0.011	
DV: Anger						224.33	<0.001	0.131
Impulsivity	0.05	0.05	0.06	25.28	<0.001			
Satisf. with teachers (ST)	−0.03	−0.05	−0.01	−2.13	0.033			
Impulsivity × ST	0.01	−0.01	0.01	0.74	0.459			
Δ*R*^2^ = 0.001						0.55	0.459	
DV: Anger^a^						116.94	<0.001	0.129
Impulsivity	0.05	0.05	0.06	17.83	<0.001			
Satisf. with teachers (ST)	−0.05	−0.08	−0.01	−2.77	0.006			
Impulsivity × ST	0.01	−0.01	0.01	0.56	0.574			
Δ*R*^2^ = 0.001						0.32	0.574	
DV: Self-harm						479.97	<0.001	0.202
Impulsivity	0.24	0.22	0.26	24.29	<0.001			
Emotion regulation (ER)	−0.26	−0.29	−0.23	−18.23	<0.001			
Impulsivity × ER	−0.03	−0.04	−0.03	−10.91	<0.001			
Δ*R*^2^ = 0.017						119.01	<0.001	
DV: Self-harm						288.57	<0.001	0.171
Impulsivity	0.24	0.22	0.26	21.21	<0.001			
Parental attachment (PA)	−0.03	−0.03	−0.03	−12.65	<0.001			
Impulsivity × PA	−0.01	−0.01	−0.01	−6.46	<0.001			
Δ*R*^2^ = 0.008						41.67	<0.001	
DV: Self-harm						225.15	<0.001	0.152
Impulsivity	0.27	0.24	0.29	22.04	<0.001			
Soc. group attach. (SGA)	−0.50	−0.65	−0.35	−6.49	<0.001			
Impulsivity × SGA	−0.10	−0.13	−0.07	−6.14	<0.001			
Δ*R*^2^ = 0.009						37.76	<0.001	
DV: Self-harm						307.61	<0.001	0.140
Impulsivity	0.29	0.27	0.31	29.47	<0.001			
Satisf. with teachers (ST)	−0.17	−0.27	−0.06	−3.16	0.002			
Impulsivity × ST	0.01	0.01	0.03	1.01	0.311			
Δ*R*^2^ = 0.001						1.03	0.311	
DV: Self-harm^a^						178.20	<0.001	0.150
Impulsivity	0.30	0.27	0.32	22.12	<0.001			
Satisf. with teachers (ST)	−0.22	−0.37	−0.08	−3.02	0.003			
Impulsivity × ST	0.01	−0.02	0.03	0.36	0.716			
Δ*R*^2^ = 0.001						0.13	0.716	
DV: Other high-risk						475.99	<0.001	0.201
Impulsivity	0.15	0.13	0.16	20.36	<0.001			
Emotion regulation (ER)	−0.22	−0.24	−0.20	−20.56	<0.001			
Impulsivity × ER	−0.03	−0.04	−0.03	−13.82	<0.001			
Δ*R*^2^ = 0.027						190.87	<0.001	
DV: Other high-risk						211.50	<0.001	0.132
Impulsivity	0.16	0.14	0.18	19.03	<0.001			
Parental attachment (PA)	−0.02	−0.02	−0.01	−9.23	<0.001			
Impulsivity × PA	−0.01	−0.01	−0.01	−5.95	<0.001			
Δ*R*^2^ = 0.007						35.29	<0.001	
DV: Other high-risk						173.83	<0.001	0.123
Impulsivity	0.18	0.16	0.20	20.02	<0.001			
Soc. group attach. (SGA)	−0.23	−0.34	−0.12	−4.00	<0.001			
Impulsivity × SGA	−0.07	−0.09	−0.04	−5.62	<0.001			
Δ*R*^2^ = 0.007						31.58	<0.001	
DV: Other high-risk						247.30	<0.001	0.115
Impulsivity	0.19	0.18	0.21	26.12	<0.001			
Satisf. with teachers (ST)	−0.15	−0.23	−0.07	−3.73	<0.001			
Impulsivity × ST	0.01	−0.02	0.02	0.09	0.932			
Δ*R*^2^ = 0.001						0.01	0.932	
DV: Other high-risk^a^						116.34	<0.001	0.103
Impulsivity	0.18	0.16	0.20	17.59	<0.001			
Satisf. with teachers (ST)	−0.18	−0.29	−0.07	−3.19	0.001			
Impulsivity × ST	−0.01	−0.02	0.02	−0.22	0.825			
Δ*R*^2^ = 0.001						0.05	0.825	

*DV* Dependent variable, *Soc. group attach.* Social group attachment, *Satisf. with teachers* Satisfaction with teachers, *Other high-risk* Other high-risk behaviors, *CI* confidence interval; *LL* lower limit, *UL* upper limit.

^a^Regression models in the younger (i.e., 13–15) adolescents’ subsample.

## References

[CR1] Allen V, Rahman N, Weissman A, MacCann C, Lewis C, Roberts RD (2015). The situational test of emotional management—brief (STEM-B): development and validation using item response theory and latent class analysis. Personality and Individual Differences.

[CR2] Amaral-Bastos M, Araújo B, Castro Caldas A (2015). Adaptação e validação da Escala Toulousiana de Coping a adolescentes. Revista Portuguesa de Enfermagem de Saúde Mental.

[CR3] Armsden GC, Greenberg MT (1987). The Inventory of Parent and Peer Attachment: Individual differences and their relationship to psychological well-being in adolescence. Journal of Youth and Adolescence.

[CR4] Assari S, Caldwell C, Mincy R (2018). Family socioeconomic status at birth and youth impulsivity at age 15; Blacks’ diminished return. Children.

[CR5] Barahmand U, Khazaee A, Hashjin GS (2016). Emotion dysregulation mediates between childhood emotional abuse and motives for substance use. Archives of Psychiatric Nursing.

[CR6] Barreto Carvalho C, Nunes C, Castilho P, da Motta C, Caldeira S, Pinto-Gouveia J (2015). Mapping non suicidal self-injury in adolescence: Development and confirmatory factor analysis of the Impulse, Self-harm and Suicide Ideation Questionnaire for Adolescents (ISSIQ-A).. Psychiatry Research.

[CR7] Barreto Carvalho, C., Cabral, J. M., Pereira, C., Cordeiro, F., Costa, R., & Arroz, A. M. (2023a). Emotion regulation weakens the associations between adverse childhood experiences and adolescent self-harm. [Manuscript submitted for publication]. Faculty of Social and Human Sciences, University of the Azores.

[CR8] Barreto Carvalho, C., Teixeira, M., Costa, R., Cordeiro, F., & Cabral, J. M. (2023b). The enhancing role of emotion regulation in the links between early positive memories and self-harm and suicidal ideation in adolescence. *Journal of Youth and Adolescence*, *52*(8), 1738–1752. 10.1007/s10964-023-01777-8. 10.1007/s10964-023-01777-8 PMC10275796 37178280

[CR9] Bentler PM (1990). Comparative fit indexes in structural models. Psychological Bulletin.

[CR10] Bolger MA, Meldrum RC, Liu L (2022). Maternal low self-control, maternal attachment toward children, parenting practices, and adolescent low self-control: A prospective 15-year study. Journal of Developmental and Life-Course Criminology.

[CR11] Bozzini AB, Bauer A, Maruyama J, Simões R, Matijasevich A (2021). Factors associated with risk behaviors in adolescence: A systematic review. Brazilian Journal of Psychiatry.

[CR12] Buss AH, Perry M (1992). The Aggression Questionnaire. Journal of Personality and Social Psychology.

[CR13] Cao Q, An J (2020). Boredom proneness and aggression among people with substance use disorder: The mediating role of trait anger and impulsivity. Journal of Drug Issues.

[CR14] Cassels M, Neufeld S, van Harmelen AL, Goodyer I, Wilkinson P (2022). Prospective pathways from impulsivity to non-suicidal self-injury among youth. Archives of Suicide Research.

[CR15] Chein J, Albert D, O’Brien L, Uckert K, Steinberg L (2011). Peers increase adolescent risk taking by enhancing activity in the brain’s reward circuitry: Peer influence on risk taking. Developmental Science.

[CR16] Crone EA, Dahl RE (2012). Understanding adolescence as a period of social-affective engagement and goal flexibility. Nature Reviews Neuroscience.

[CR17] Cunha, M., Xavier, A., Martinho, M. I., & Matos, M. (2014). Measuring positive emotional memories in adolescents: Psychometric properties and confirmatory factor analysis of the Early Memories of Warmth and Safeness Scale. *International Journal of Psychology and Psychological Therapy*, *14*(2), 245–259. https://www.ijpsy.com/volumen14/num2/386.html.

[CR45] da Motta C, Carvalho CB, Pato MT, Castilho P (2021). Rasch measurement of the brief situational test of emotional management in a large Portuguese sample. Journal of Psychoeducational Assessment.

[CR18] Dahl RE, Allen NB, Wilbrecht L, Suleiman AB (2018). Importance of investing in adolescence from a developmental science perspective. Nature.

[CR19] Decker JH, Lourenco FS, Doll BB, Hartley CA (2015). Experiential reward learning outweighs instruction prior to adulthood. Cognitive, Affective, & Behavioral Neuroscience.

[CR20] Dinis, A., Matos, M., & Pinto Gouveia, J. (2008). *Validação da Escala de Vinculação ao Grupo Social na população portuguesa* [Validation of the Social Group Attachment Scale in the Portuguese population] [Unpublished manuscript]. University of Coimbra.

[CR21] Esparbès, S., Sordes-Ader, F., & Tap, P. (1993). Présentation de l’échelle de coping. In *Actes de las Journées du Laboratoire Personnalisation et Changements Sociaux* (pp. 89–107).

[CR22] Fabrigar LR, Wegener DT, MacCallum RC, Strahan EJ (1999). Evaluating the use of exploratory factor analysis in psychological research. Psychological Methods.

[CR23] Felgueiras MC, Festas C, Vieira M (2010). Adaptação e validação da Resilience Scale® de Wagnild e Young para a cultura portuguesa. Cadernos de Saúde.

[CR24] Ferguson CJ (2009). An effect size primer: A guide for clinicians and researchers. Professional Psychology: Research and Practice.

[CR25] Gottfredson, M. R., & Hirschi, T. (1990). *A general theory of crime*. Redwood City, CA: Stanford University Press.

[CR26] Hasking P, Claes L (2020). Transdiagnostic mechanisms involved in nonsuicidal self-injury, risky drinking and disordered eating: Impulsivity, emotion regulation and alexithymia. Journal of American College Health.

[CR27] Hayes, A. F. (2013). *Introduction to mediation, moderation, and conditional process analysis: A regression-based approach.* New York, NY: Guilford Press.

[CR28] Hentges RF, Shaw DS, Wang MT (2018). Early childhood parenting and child impulsivity as precursors to aggression, substance use, and risky sexual behavior in adolescence and early adulthood. Development and Psychopathology.

[CR29] Hessler DM, Katz LF (2010). Brief report: Associations between emotional competence and adolescent risky behavior. Journal of Adolescence.

[CR30] Icenogle G, Cauffman E (2021). Adolescent decision making: A decade in review. Journal of Research on Adolescence.

[CR31] Inuggi A, Sanz-Arigita E, González-Salinas C, Valero-García AV, García-Santos JM, Fuentes LJ (2014). Brain functional connectivity changes in children that differ in impulsivity temperamental trait. Frontiers in Behavioral Neuroscience.

[CR32] Jelihovschi APG, Cardoso RL, Linhares A (2018). An analysis of the associations among cognitive impulsiveness, reasoning process, and rational decision making. Frontiers in Psychology.

[CR33] Johnston C, Mash EJ (2001). Families of children with attention-deficit/hyperactivity disorder: Review and recommendations for future research. Clinical Child and Family Psychology Review.

[CR34] Joo YS, Lee WK (2020). Does living in a chaotic home predict adolescent delinquency? A moderated mediation model of impulsivity and school connectedness. Children and Youth Services Review.

[CR35] Khurana, A., & Romer, D. (2020). Impulsivity in adolescence: Predictors and consequences. In S. Hupp & J. D. Jewell (Eds.), *The Encyclopedia of Child and Adolescent Development*. 10.1002/9781119171492.wecad472

[CR36] Kipping RR, Campbell RM, MacArthur GJ, Gunnell DJ, Hickman M (2012). Multiple risk behaviour in adolescence. Journal of Public Health.

[CR37] Krumpal I (2013). Determinants of social desirability bias in sensitive surveys: A literature review. Quality and Quantity.

[CR38] Li Q, Dai W, Zhong Y, Wang L, Dai B, Liu X (2019). The mediating role of coping styles on impulsivity, behavioral inhibition/approach system, and internet addiction in adolescents from a gender perspective. Frontiers in Psychology.

[CR39] Liu W, Lee GP, Goldweber A, Petras H, Storr CL, Ialongo NS (2013). Impulsivity trajectories and gambling in adolescence among urban male youth. Addiction.

[CR40] Löbler ML, Reis E, Nishi JM, Tagliapietra RD (2019). Decision-making styles inventory: Instrument validation in the Brazilian context.. Revista de Administração da UNIMEP.

[CR41] Machado TS, Oliveira M (2007). Vinculação aos pais em adolescentes portugueses: O estudo de Coimbra [Parental attachment in Portuguese adolescents: The Coimbra study]. Psicologia e Educação.

[CR42] MacPherson HA, Kim KL, Seymour KE, Wolff J, Esposito-Smythers C, Spirito A, Dickstein DP (2022). Cognitive flexibility and impulsivity deficits in suicidal adolescents. Research on Child and Adolescent Psychopathology.

[CR43] Menting B, van Lier PAC, Koot HM, Pardini D, Loeber R (2015). Cognitive impulsivity and the development of delinquency from late childhood to early adulthood: Moderating effects of parenting behavior and peer relationships. Development and Psychopathology.

[CR44] Mischel W, Ayduk O, Berman MG, Casey BJ, Gotlib IH, Jonides J, Kross E, Teslovich T, Wilson NL, Zayas V, Shoda Y (2011). ‘Willpower’ over the life span: Decomposing self-regulation. Social Cognitive and Affective Neuroscience.

[CR46] Moustafa AA, Tindle R, Frydecka D, Misiak B (2017). Impulsivity and its relationship with anxiety, depression and stress. Comprehensive Psychiatry.

[CR47] Moyano N, Vélez K, Arias A, del Mar Sánchez-Fuentes M (2022). Two pathways to suicidal intention in Ecuadorian adolescents: The role of parental and peer attachment, depression and impulsivity. Current Psychology.

[CR48] Perez NM, Jennings WG, Piquero AR, Baglivio MT (2016). Adverse childhood experiences and suicide attempts: The mediating influence of personality development and problem behaviors. Journal of Youth and Adolescence.

[CR49] Quintana-Orts C, Mérida-López S, Rey L, Neto F, Extremera N (2020). Untangling the emotional intelligence-suicidal ideation connection: The role of cognitive emotion regulation strategies in adolescents. Journal of Clinical Medicine.

[CR50] Ragan, D. T., Osgood, D. W., & Kreager, D. A. (2022). Impulsivity, peers, and delinquency: A dynamic social network approach. *Journal of Quantitative Criminology*. Advance online publication. 10.1007/s10940-022-09547-8 10.1007/s10940-022-09547-8 PMC13148287 42099894

[CR51] Ran H, Fang D, Che Y, Donald AR, Peng J, Chen L, Wang S, Xiao Y (2022). Resilience mediates the association between impulsivity and self-harm in Chinese adolescents. Journal of Affective Disorders.

[CR52] Richter A, Gilbert P, McEwan K (2009). Development of an early memories of warmth and safeness scale and its relationship to psychopathology. Psychology and Psychotherapy: Theory, Research and Practice.

[CR53] Rosenbaum, G. M., & Hartley, C. A. (2019). Developmental perspectives on risky and impulsive choice. *Philosophical Transactions of the Royal Society B: Biological Sciences*, *374*(1766), Article 20180133. 10.1098/rstb.2018.0133 10.1098/rstb.2018.0133 PMC6335462 30966918

[CR54] Sameroff A (2010). A unified theory of development: A dialectic integration of nature and nurture. Child Development.

[CR55] Scott LN, Levy KN, Pincus AL (2009). Adult attachment, personality traits, and borderline personality disorder features in young adults. Journal of Personality Disorders.

[CR56] Scott SG, Bruce RA (1995). Decision-making style: The development and assessment of a new measure. Educational and Psychological Measurement.

[CR57] Shulman EP, Smith AR, Silva K, Icenogle G, Duell N, Chein J, Steinberg L (2016). The dual systems model: Review, reappraisal, and reaffirmation. Developmental Cognitive Neuroscience.

[CR58] Simões A (1993). São os homens mais agressivos que as mulheres?. Revista Portuguesa de Pedagogia.

[CR59] Smith ER, Murphy J, Coats S (1999). Attachment to groups: Theory and measurement. Journal of Personality and Social Psychology.

[CR60] Song S, Marcum CS, Wilkinson AV, Shete S, Koehly LM (2019). Genetic, psychological, and personal network factors associated with changes in binge drinking over 2 years among Mexican heritage adolescents in the USA. Annals of Behavioral Medicine.

[CR61] Steinberg L (2008). A social neuroscience perspective on adolescent risk-taking. Developmental Review.

[CR62] Steinberg L, Albert D, Cauffman E, Banich M, Graham S, Woolard J (2008). Age differences in sensation seeking and impulsivity as indexed by behavior and self-report: evidence for a dual systems model. Developmental Psychology.

[CR63] Stoyanova SY, Ivantchev N (2021). Associations among functional and dysfunctional impulsivity: Direct and indirect effects on sensation seeking in youth (19-25 years old).. Psychological Thought.

[CR64] Turiuc MN, Pojoga S (2018). Reward, impulsivity and parental self-control as antecedents of self-control in children. Annals of the Alexandru Ioan Cuza University Psychology Series.

[CR65] VanderVeen JD, Hershberger AR, Cyders MA (2016). UPPS-P model impulsivity and marijuana use behaviors in adolescents: A meta-analysis. Drug and Alcohol Dependence.

[CR66] Wagnild GM, Young H (1993). Development and psychometric evaluation of the resilience scale. Journal of Nursing Measurement.

[CR67] Whiteside SP, Lynam DR (2001). The Five Factor Model and impulsivity: Using a structural model of personality to understand impulsivity. Personality and Individual Differences.

[CR68] Wills TA, Bantum EOC (2012). Social support, self-regulation, and resilience in two populations: General-population adolescents and adult cancer survivors. Journal of Social and Clinical Psychology.

[CR69] World Health Organization. (n.d.). *Adolescent health*. www.who.int/health-topics/adolescent-health

[CR70] Zanus C, Battistutta S, Aliverti R, Monasta L, Montico M, Ronfani L, Carrozzi M (2021). High-school students and self-injurious thoughts and behaviours: Clues of emotion dysregulation. Italian Journal of Pediatrics.

[CR71] Zhang J, Lin L (2015). The moderating effect of social support on the relationship between impulsivity and suicide in rural China. Community Mental Health Journal.

[CR72] Zuckerman, M. (1994). *Behavioral expressions and biosocial bases of sensation seeking*. Cambridge University Press.

