# Supplementary material for: “Help Me Control My Impulses!”: Adolescent Impulsivity and Its Negative Individual, Family, Peer, and Community Explanatory Factors
Source: J Youth Adolesc. 2023 Aug 24;52(12):2545–58. doi: 10.1007/s10964-023-01837-z (PMC10522498; doi:10.1007/s10964-023-01837-z)
Supplement: Supplementary file 1 — Supplementary Material [file 10964_2023_1837_MOESM1_ESM.docx]

**“Help me Control my Impulses!”: Adolescent Impulsivity and its Negative Individual, Family, Peer, and Community Explanatory Factors**

**Supplementary Material**

Supplementary Text 1. Description of Measures

Supplementary Text 2. Description of Simple Slopes Analyses

Supplementary Text 3. Description of Sensitivity Analyses

Supplementary Table 1. Pearson Correlation Coefficients between Impulsivity and Sociodemographic, Early Relationships, Personological, Self-regulatory, and Social Relationships Variables by Specific Age Group

Supplementary Table 2. Results of the Hierarchical Multiple Linear Regression of Impulsivity by Specific Age Group

Supplementary Table 3. Pearson Correlation Coefficients between Impulsivity and Sociodemographic, Early Relationships, Personological, Self-regulatory, and Social Relationships Variables by Specific Age Group

Supplementary Table 4. Results of the Hierarchical Multiple Linear Regression of Impulsivity in Younger (13-15) and Older (16-19) Adolescents by Gender

Supplementary Text 1. Description of Measures

**Measures**

**Early Memories of Warmth and Safeness**

The Early Memories of Warmth and Safeness for Adolescents Scale (EMWSS-A; original version by Richard et al., 2009; Portuguese version by Cunha et al., 2014) was used to measure personal emotional memories related to being cared about in childhood. It is a self-report scale comprising 21 items (e.g., “I felt secure and safe”) scored on a 5-point Likert scale ranging from 0 = *No, never* to 4 = *Yes, most of the time*. In the original study (Richard et al., 2009), in the Portuguese version (Cunha et al., 2014), and in the current study, the scale showed excellent internal consistency, α = .97, .95, and .98, respectively.

**Rational Decision-making Style**

The General Decision-Making Style Scale (GDMSS; original version by Scott & Bruce, 1995; Brazilian version by Löbler et al., 2019) was used to assess rational decision-making style. It is a self-report measure comprising 25 items, scored on a 5-point Likert scale ranging from 1 = *Strongly disagree* to 5 = *Strongly agree*, grouped into five decision-making styles: rational (five items; e.g., “My decision-making requires careful thought”), intuitive (five items; e.g., “When I make decisions, I tend to rely on my intuition”), dependent (five items; e.g., “I rarely make important decisions without consulting other people”), avoidant (five items; e.g., “I postpone decision-making whenever possible”), and spontaneous (five items; e.g., “I often make decisions on the spur of the moment”). In the original version (Scott & Bruce, 1995), the scale dimensions showed acceptable to excellent internal consistency, α = .68 and .94. In the Brazilian version (Löbler et al., 2019), it showed acceptable to good internal consistency, α = .62 for rational and .79 for dependent. In this study, the subscales presented good internal consistency, α = .77 for rational and .83 for spontaneous.

**Resilience**

The Resilience Scale (RS; original version by Wagnild & Young, 1993; Portuguese version by Felgueiras et al., 2010) was used to measure resilience. It is a self-report measure comprising 24 items, scored on a Likert scale ranging from 1 = *Strongly disagree* to 7 = *Strongly agree*, grouped into five dimensions derived from the original qualitative study: perseverance (six items; e.g., “I am determined”), self-reliance (seven items; e.g., “I have self-discipline”), equanimity (four items; e.g., “I live one day at a time”), meaningfulness (five items; e.g., “My life has meaning”), existential aloneness (two items; e.g., “I am more able to depend on myself than on any other person”). In this study, only the overall resilience score was used. In the original study (Wagnild & Young, 1993), the overall scale showed excellent internal consistency, α = .91. In the Portuguese version (Felgueiras et al., 2010), it showed good internal consistency, α = .82. In this study, the overall scale showed excellent internal consistency, α = .94.

**Emotion Regulation**

The Situational Test of Emotional Management-Brief (STEM-B; original version by Allen et al., 2015; Portuguese version by da Motta et al., 2021) was used to measure emotion regulation. It comprises 19 items describing hypothetical scenarios (e.g., “Joana and Marina shared an office for years, but Joana got a new job and Marina lost touch with her”) in which individuals, out of four responses, choose the one they believe to be the most effective way to deal with the situation (e.g., “Simply accept that Joana is gone and the friendship is over”, “Call Joana and invite her to lunch or drink a coffee to catch up”, “Contact Joana and invite her for a chat but also make friends with the person who replaced her at the office”, “Get to know other people at the office and make new friendships”). Dichotomous scoring is used, with the most appropriate answer being scored as 1 and the remaining options as 0. In the original version (Allen et al., 2015), this scale displayed good internal consistency, α = .84. In the Portuguese version (da Motta et al., 2021) and in this study, it showed acceptance internal consistency, α = .62 and .60, respectively.

**Coping**

Coping was examined using the Toulousiana Coping Scale (TCS; original version by Esparbès et al., 1993; Portuguese adolescent version by Amaral-Bastos et al., 2015), a self-report measure comprising 51 items scored on a 5-point Likert scale ranging from 1 = *Never* to 5 = *Always* and grouped into 5 subscales: control (14 items; e.g., “I accept the problem if it is unavoidable”); social support (11 items; e.g., “I discuss my problem with my parents”); withdrawal, conversion, and additivity (14 items; e.g., “I change my behavior”); social distraction (six items; e.g., “I try not to think of the problem”); and refusal (six items; e.g., “I react as if the problem did not exist”). In this study, only the total scale was used. In the Portuguese version (Amaral-Bastos et al., 2015), the overall scale showed good internal consistency, α = .85. In this study, it showed excellent internal consistency, α = .96.

**Parental Attachment**

The Inventory of Parent and Peer Attachment (IPPA; original version by Armsden & Greenberg, 1987; Portuguese version by Machado & Oliveira, 2007) was used to assess parental attachment. It is a self-report measure comprising 28 items, answered first in relation to the maternal figure and then in relation to the paternal figure, scored on a 5-point Likert scale ranging from 1 = *Almost never or never true* to 5 = *Almost always or always true*. The items are grouped into three dimensions: parental trust (10 items; e.g., “My mother/father accepts me as I am”), parental communication (10 items; e.g., “I tell my mother/father about my problems and troubles”), and parental alienation (8 items; e.g., “I don’t get much attention from my mother/father”). In this study only the overall score of parental attachment was computed. In the original study (Armsden & Greenberg, 1987), the overall scale showed good internal consistency, α = .87 for maternal attachment and .89 for paternal attachment. In the Portuguese version (Machado & Oliveira, 2007), it also showed good internal consistency, α = .87. In this study, the overall scale exhibited excellent internal consistency, α = .92.

**Social Group Attachment**

The Social Group Attachment Scale (SGAS; original version by Smith et al., 1999; Portuguese version by Dinis et al., 2008) was used to measure experiences and feelings of attachment toward the social group participants consider to be the most important. It is a 25-item (e.g., “I do not often worry about being abandoned by my group”) scale scored on a Likert scale ranging from 1 = *Strongly disagree* to 7 = *Strongly agree*. Multiple items are grouped into two 10-item dimensions of insecure attachment, anxious and avoidant; however, in the present study, only the total scale (i.e., secure social group attachment) was used because it is the only one that may decrease the risk for impulsivity. In the original study (Smith et al., 1999), this scale showed acceptable to good internal consistency, α = .75 for avoidant attachment and .86 for anxious attachment. In this study, it showed acceptable internal consistency, α = .79.

**Satisfaction with School-related Variables**

Participants were asked to indicate their degree of satisfaction with some school-related aspects (i.e., school, classmates, peers from other classes, friends from school, teachers, staff) using six items starting with “How satisfied are you with your…?” scored on a 5-point Likert scale ranging from 1 = *Very unsatisfied* to 5 = *Very satisfied*.

**Family Satisfaction**

Adolescents were asked to indicate their level of satisfaction with some family members (i.e., parents, siblings, remaining family) using three items starting with “How satisfied are you with your...?” scored on a 5-point Likert scale ranging from 1 = *Very unsatisfied* to 5 = *Very satisfied*.

**Aggression, Self-harm, and Other High-risk Behaviors**

To examine verbal aggression, anger, self-harm, and other high-risk behaviors (e.g., substance use, reckless driving, risky sexual behavior), adolescents completed the Buss-Perry Aggression Questionnaire (AQ; original version by Buss & Perry, 1992; Portuguese version by Simões, 1993) and the ISSIQ-A (Barreto Carvalho et al., 2015; described above). The AQ is comprised of 29 items scored on a Likert scale ranging from 1 = *Never or almost never* and 5 = *Always or almost always* and grouped into four components of aggression: physical aggression (nine items; e.g., “I get into fights than the average person”), verbal aggression (five items; e.g., “I can’t help getting into arguments when people disagree with me”), anger (seven items; e.g., “Sometimes I fly off the handle for no good reason”), and hostility (eight items; e.g., “I wonder why sometimes I feel so bitter about things”). In this study, only the verbal aggression and the anger subscales were used. In the original version of the AQ (Buss & Perry, 1992), all dimensions revealed acceptable to good internal consistencies, αs ranging from .72 for verbal aggression to .85 for physical aggression. Similarly, the Portuguese version (Simões, 1993) showed acceptable to good internal consistencies, αs ranging from .60 for verbal aggression and .87 for the overall scale. In this study, verbal aggression and anger showed acceptable internal consistencies, αs = .73 and .75, respectively. In the validation study of the ISSIQ-A (Barreto Carvalho et al., 2015), the self-harm and the other high-risk behaviors subscales showed excellent and good internal consistencies, α = .90 and α = .81, respectively. In this study, they showed excellent internal consistencies, α = .95 for self-harm and .97 for other high-risk behaviors.

Supplementary Text 2. Description of Simple Slopes Analyses

At lower than average levels of emotion regulation, adolescents perceived a greater effect of impulsivity on self-harm, *B* = 0.34, *p* < .001, and other high-risk behaviors, *B* = 0.24, *p* < .001, compared to at average, *B* = 0.24, *p* < .001, for self-harm and *B* = 0.15, *p* < .001, for other high-risk behaviors, and at higher than average levels, *B* = 0.14, *p* < .001, for self-harm and *B* = 0.06, *p* < .001, for other high-risk behaviors. Similarly, at lower than average levels of parental attachment, they perceived a greater effect of impulsivity on self-harm, *B* = 0.31, *p* < .001, and other high-risk behaviors, *B* = 0.21, *p* < .001, compared to at average, *B* = 0.24, *p* < .001, for self-harm and *B* = 0.16, *p* < .001, for other high-risk behaviors, and at higher than average levels, *B* = 0.17, *p* < .001, for self-harm and *B* = 0.11, *p* < .001, for other high-risk behaviors. Lastly, at lower than average levels of the social group attachment, participants experienced a greater effect of impulsivity on verbal aggression, *B* = 0.05, *p* < .001, anger, *B* = 0.05, *p* < .001, self-harm, *B* = 0.33, *p* < .001, and other high-risk behaviors, *B* = 0.23, *p* < .001, compared to at average, *B* = 0.04, *p* < .001, for verbal aggression, *B* = 0.04, *p* < .001, for anger, *B* = 0.27, *p* < .001, for self-harm, and *B* = 0.18, *p* < .001, for other high-risk behaviors, and at higher than average levels, *B* = 0.04, *p* < .001, for verbal aggression, *B* = 0.04, *p* < .001, for anger, *B* = 0.20, *p* < .001, for self-harm, and *B* = 0.14, *p* < .001, for other high-risk behaviors.

Supplementary Text 3. Description of Sensitivity Analyses

Correlations were conducted between impulsivity and the variables hypothesized to negatively associate with this trait by age (i.e., 13, 14, 15, 16, 17, 18 or older). Across all ages, impulsivity was negatively associated with emotion regulation, parental attachment, social group attachment, and with satisfaction with teachers. Additionally, in the 13 and the 16-year-old adolescents, impulsivity was negatively linked with gender; across all ages except for the 16-year-olds, it was negatively associated with early memories of warmth and safeness; across all ages except for the 13-year-olds, it was negatively linked with satisfaction with parents; in the 13 and the 14-year-olds, impulsivity was negatively linked with satisfaction with school staff and remaining family; impulsivity was also negatively associated with satisfaction with remaining family in the 17-year-olds; in the adolescents who are 15, 17, and 18 or older, it was negatively associated with rational decision-making style; in the 13, 14, 15, and 16-year-olds, it was negatively linked with school satisfaction; in the 13 and the 14-year-olds, it was negatively associated with satisfaction with siblings; in the 13, 14, and 16-year-olds, impulsivity was negatively linked with satisfaction with classmates; impulsivity was negatively associated with satisfaction with friends from school only in the 14-year-olds; lastly, it was negatively linked with resilience only in the 15-year-olds. To explore the specific negative correlates/protective factors of impulsivity in each age group (i.e., 13, 14, 15, 16, 17, 18 or older), six multiple regressions – one for each age group – using the stepwise method were conducted using the corresponding variables that correlated negatively with impulsivity as the explanatory variables. At each step, explanatory variables were chosen based on *p*-values (*p* ≤ .05 for entry and ≥ .10 for removal). To increase readability, only the significant results present in the last models of each regression are summarized below.

Parental attachment had a negative effect on impulsivity in all ages except for those who are 18 or older; social group attachment had this effect across all ages except for the 17-year-olds; emotion regulation had a negative effect on impulsivity in the adolescents who are 14, 16, 17, and 18 or older; rational decision-making style had this effect only in those 18 or older; lastly, satisfaction with teachers had this effect only in the 14-year-olds.

Additionally, correlations were conducted between impulsivity and all other variables by gender in younger (i.e., 13-15) and older (i.e., 16-19) adolescents. Across all groups, impulsivity was negatively associated with emotion regulation, parental attachment, social group attachment, and satisfaction with teachers; in the younger adolescents of both genders and in the older females, impulsivity was negatively linked with early memories of warmth and safeness and satisfaction with school staff; only in the younger adolescents of both genders, impulsivity was negatively linked with school satisfaction; it was negatively linked with rational decision-making style, and satisfaction with classmates, parents, siblings, and remaining family in both younger and older females; lastly, impulsivity was negatively linked with resilience only in the younger females. To explore the specific negative correlates/protective factors of impulsivity by gender in younger and older adolescents, four multiple regressions – one for each age group (i.e., younger females, older females, younger males, older females) – using the stepwise method were conducted using the corresponding variables that correlated negatively with impulsivity as the explanatory variables. At each step, explanatory variables were chosen based on *p*-values (*p* ≤ .05 for entry and ≥ .10 for removal). To increase readability, only the significant results present in the last models of each regression are summarized below.

Parental attachment and social group attachment had a negative effect on impulsivity in all groups; emotion regulation had this effect in the older adolescents of both genders and in the younger males; only in the younger females, satisfaction with teachers had a negative effect on impulsivity; lastly, rational decision-making style had this effect only in the older females.

Supplementary Table 1. Pearson Correlation Coefficients between Impulsivity and Possible Negative Correlates and Protective Factors by Specific Age Group

|  | Impulsivity | |
| --- | --- | --- |
|  | *n* | *r* |
| 13-year-olds |  |  |
| Gender | 909 | -.12^***^ |
| Early memories of warmth and safeness | 875 | -.20^***^ |
| Rational decision-making style | 669 | -.04 |
| Resilience | 812 | -.06 |
| Emotion regulation | 907 | -.17^***^ |
| Coping | 686 | .15^***^ |
| Parental attachment | 681 | -.28^***^ |
| Social group attachment | 589 | -.28^***^ |
| School satisfaction | 907 | -.10^**^ |
| Satisfaction with classmates | 907 | -.07^*^ |
| Satisfaction with peers from other classes | 908 | -.02 |
| Satisfaction with friends from school | 908 | -.01 |
| Satisfaction with friends from outside of school | 908 | -.04 |
| Satisfaction with teachers | 909 | -.13^***^ |
| Satisfaction with school staff | 909 | -.12^***^ |
| Satisfaction with parents | 908 | -.06 |
| Satisfaction with siblings | 823 | -.11^**^ |
| Satisfaction with remaining family | 834 | -.07^*^ |
| 14-year-olds |  |  |
| Gender | 1,064 | .01 |
| Early memories of warmth and safeness | 1,037 | -.10^**^ |
| Rational decision-making style | 772 | -.07 |
| Resilience | 943 | -.01 |
| Emotion regulation | 1,064 | -.17^***^ |
| Coping | 781 | .15^***^ |
| Parental attachment | 785 | -.23^***^ |
| Social group attachment | 683 | -.24^***^ |
| School satisfaction | 1,062 | -.13^***^ |
| Satisfaction with classmates | 1,064 | -.08^*^ |
| Satisfaction with peers from other classes | 1,061 | -.03 |
| Satisfaction with friends from school | 1,061 | -.08^**^ |
| Satisfaction with friends from outside of school | 1,062 | -.02 |
| Satisfaction with teachers | 1,064 | -.18^***^ |
| Satisfaction with school staff | 1,063 | -.13^***^ |
| Satisfaction with parents | 1,061 | -.08^*^ |
| Satisfaction with siblings | 981 | -.08^*^ |
| Satisfaction with remaining family | 994 | -.10^**^ |
| 15-year-olds |  |  |
| Gender | 1,359 | .01 |
| Early memories of warmth and safeness | 1,324 | -.12^***^ |
| Rational decision-making style | 920 | -.11^***^ |
| Resilience | 1,097 | -.07^*^ |
| Emotion regulation | 1,358 | -.14^***^ |
| Coping | 929 | .12^***^ |
| Parental attachment | 931 | -.30^***^ |
| Social group attachment | 814 | -.20^***^ |
| School satisfaction | 1,354 | -.13^***^ |
| Satisfaction with classmates | 1,351 | -.01 |
| Satisfaction with peers from other classes | 1,355 | .02 |
| Satisfaction with friends from school | 1,351 | .01 |
| Satisfaction with friends from outside of school | 1,353 | .09^**^ |
| Satisfaction with teachers | 1,358 | -.15^***^ |
| Satisfaction with school staff | 1,357 | -.03 |
| Satisfaction with parents | 1,353 | -.06^*^ |
| Satisfaction with siblings | 1,272 | -.03 |
| Satisfaction with remaining family | 1,270 | -.01 |
| 16-year-olds |  |  |
| Gender | 1,116 | -.06^*^ |
| Early memories of warmth and safeness | 1,084 | -.04 |
| Rational decision-making style | 792 | -.06 |
| Resilience | 962 | .07^*^ |
| Emotion regulation | 1,117 | -.24^***^ |
| Coping | 800 | .12^***^ |
| Parental attachment | 807 | -.24^***^ |
| Social group attachment | 744 | -.26^***^ |
| School satisfaction | 1,117 | -.07^*^ |
| Satisfaction with classmates | 1,118 | -.06^*^ |
| Satisfaction with peers from other classes | 1,116 | .05 |
| Satisfaction with friends from school | 1,115 | .03 |
| Satisfaction with friends from outside of school | 1,116 | .04 |
| Satisfaction with teachers | 1,118 | -.10^**^ |
| Satisfaction with school staff | 1,117 | .01 |
| Satisfaction with parents | 1,117 | -.06^*^ |
| Satisfaction with siblings | 1,055 | -.02 |
| Satisfaction with remaining family | 1,064 | -.04 |
| 17-year-olds |  |  |
| Gender | 834 | -.03 |
| Early memories of warmth and safeness | 815 | -.08^*^ |
| Rational decision-making style | 603 | -.13^***^ |
| Resilience | 745 | .01 |
| Emotion regulation | 833 | -.19^***^ |
| Coping | 617 | .10^*^ |
| Parental attachment | 617 | -.26^***^ |
| Social group attachment | 557 | -.18^***^ |
| School satisfaction | 824 | -.06 |
| Satisfaction with classmates | 832 | -.02 |
| Satisfaction with peers from other classes | 829 | -.01 |
| Satisfaction with friends from school | 831 | -.01 |
| Satisfaction with friends from outside of school | 831 | .09^**^ |
| Satisfaction with teachers | 833 | -.07^*^ |
| Satisfaction with school staff | 831 | -.05 |
| Satisfaction with parents | 833 | -.07^*^ |
| Satisfaction with siblings | 794 | -.04 |
| Satisfaction with remaining family | 791 | -.08^*^ |
| 18 or older |  |  |
| Gender | 885 | .01 |
| Early memories of warmth and safeness | 862 | -.09^**^ |
| Rational decision-making style | 683 | -.14^***^ |
| Resilience | 803 | .02 |
| Emotion regulation | 883 | -.17^***^ |
| Coping | 674 | .06 |
| Parental attachment | 690 | -.18^***^ |
| Social group attachment | 644 | -.15^***^ |
| School satisfaction | 884 | -.03 |
| Satisfaction with classmates | 885 | -.02 |
| Satisfaction with peers from other classes | 884 | -.02 |
| Satisfaction with friends from school | 885 | -.05 |
| Satisfaction with friends from outside of school | 885 | -.01 |
| Satisfaction with teachers | 884 | -.08^*^ |
| Satisfaction with school staff | 884 | -.06 |
| Satisfaction with parents | 885 | -.07^*^ |
| Satisfaction with siblings | 838 | -.04 |
| Satisfaction with remaining family | 858 | -.01 |

*Note*. Impulsivity was measured using the Impulse, Self-harm and Suicide Ideation Questionnaire for Adolescents (ISSIQ-A; Barreto Carvalho et al., 2015). Rational decision-making style was measured using the General Decision-Making Style Scale (GDMSS; Scott & Bruce, 1995; Brazilian version by Löbler et al., 2019). Resilience was measured using the Resilience Scale (RS; Wagnild & Young, 1993; Portuguese version by Felgueiras et al., 2010). Emotion regulation was measured using the Situational Test of Emotion Management-Brief (STEM-B; Allen et al., 2015; Portuguese version by da Motta et al., 2021). Coping was measured using the Toulousiana Coping Scale (TCS; Esparbès et al., 1993; Portuguese version by Amaral-Bastos et al., 2015). Parental attachment was measured using the Inventory of Parent and Peer Attachment (IPPA; Armsden & Greenberg, 1987; Portuguese version by Machado & Oliveira, 2007). Social group attachment was measured using the Social Group Attachment Scale (SGAS; Smith et al., 1999; Portuguese version by Dinis et al., 2008).

^*^*p* < .05; ^**^*p* < .01; ^***^*p* < .001

Supplementary Table 2. Results of the Stepwise Multiple Linear Regression of Impulsivity by Specific Age Group

|  | **Model 1** | | | | | **Model 2** | | | | | **Model 3** | | | | | | **Model 4** | | | | | | | |
| --- | --- | --- | --- | --- | --- | --- | --- | --- | --- | --- | --- | --- | --- | --- | --- | --- | --- | --- | --- | --- | --- | --- | --- | --- |
|  | *B* | *SE* | | β | *t* | *B* | *SE* | | β | *t* | *B* | *SE* | | β | | *t* | *B* | | *SE* | | | β | | *t* |
| 13-year-olds |  |  | |  |  |  |  | |  |  |  |  | |  | |  |  | |  | | |  | |  |
| **Independent variables** |  |  | |  |  |  |  | |  |  |  |  | |  | |  |  | |  | | |  | |  |
| Social group attachment | -1.91 | 0.30 | | -0.29 | -6.32^***^ | -1.46 | 0.31 | | -0.22 | -4.65^***^ |  |  | |  | |  |  | |  | | |  | |  |
| Parental attachment |  |  | |  |  | -0.05 | 0.01 | | -0.20 | -4.20^***^ |  |  | |  | |  |  | |  | | |  | |  |
| **Model statistics** |  | | | | |  | | | | |  | | | | | |  | | | | | | | |
| *R*^2^ | .083 | | | | | .118 | | | | |  | | | | | |  | | | | | | | |
| Δ*R*^2^ |  | | | | | .035 | | | | |  | | | | | |  | | | | | | | |
| Adj. *R*^2^ | .081 | | | | | .114 | | | | |  | | | | | |  | | | | | | | |
| *F* | 39.90^***^ | | | | | 20.53^***^ | | | | |  | | | | | |  | | | | | | | |
| Δ*F* |  | | | | | 17.66^***^ | | | | |  | | | | | |  | | | | | | | |
| 14-year-olds |  | | | | |  | | | | |  | | | | | |  | | | | | | | |
| **Independent variables** |  | | | | |  | | | | |  | | | | | |  | | | | | | | |
| Parental attachment | -0.05 | | 0.01 | -0.23 | -5.58^***^ | -0.04 | 0.01 | -0.18 | | -4.17^***^ | -0.04 | 0.01 | -0.16 | | -3.73^***^ | | -0.03 | 0.01 | | | -0.14 | | -3.09^**^ | |
| Social group attachment |  | |  |  |  | -1.12 | 0.28 | -0.17 | | -4.03^***^ | -1.09 | 0.28 | -0.17 | | -3.92^***^ | | -1.09 | 0.28 | | | -0.17 | | -3.92^***^ | |
| Satisfaction with teachers |  | |  |  |  |  |  |  | |  | -0.61 | 0.23 | -0.11 | | -2.66^**^ | | -0.54 | 0.23 | | | -0.10 | | -2.38^*^ | |
| Emotion regulation |  | |  |  |  |  |  |  | |  |  |  |  | |  | | -0.16 | 0.07 | | | -0.10 | | -2.29^*^ | |
| **Model statistics** |  | | | | |  | | | | |  | | | | | |  | | | | | | | |
| *R*^2^ | .054 | | | | | .082 | | | | | .093 | | | | | | .102 | | | | | | | |
| Δ*R*^2^ |  | | | | | .027 | | | | | .012 | | | | | | .009 | | | | | | | |
| Adj. *R*^2^ | .052 | | | | | .078 | | | | | .088 | | | | | | .095 | | | | | | | |
| *F* | 31.09^***^ | | | | | 24.10^***^ | | | | | 18.60^***^ | | | | | | 15.37^***^ | | | | | | | |
| Δ*F* |  | | | | | 16.24^***^ | | | | | 7.05^**^ | | | | | | 5.26^*^ | | | | | | | |
| 15-year-olds |  | | | | |  | | | | |  | | | | | |  | | | | | | | |
| **Independent variables** |  | | | | |  | | | | |  | | | | | |  | | | | | | | |
| Parental attachment | -0.07 | | 0.01 | -0.31 | -8.20^***^ | -0.06 | 0.01 | -0.28 | | -7.41^***^ | -0.06 | 0.01 | -0.25 | | -6.22^***^ | |  |  | | |  | |  | |
| Satisfaction with teachers |  | |  |  |  | -0.78 | 0.22 | -0.14 | | -3.57^***^ | -0.76 | 0.22 | -0.13 | | -3.47^***^ | |  |  | | |  | |  | |
| Social group attachment |  | |  |  |  |  |  |  | |  | -0.66 | 0.26 | -0.10 | | -2.52^*^ | |  |  | | |  | |  | |
| **Model statistics** |  | | | | |  | | | | |  | | | | | |  | | | | | | | |
| *R*^2^ | .094 | | | | | .112 | | | | | .120 | | | | | |  | | | | | | | |
| Δ*R*^2^ |  | | | | | .018 | | | | | .009 | | | | | |  | | | | | | | |
| Adj. *R*^2^ | .093 | | | | | .109 | | | | | .116 | | | | | |  | | | | | | | |
| *F* | 67.15^***^ | | | | | 40.57^***^ | | | | | 29.40^***^ | | | | | |  | | | | | | | |
| Δ*F* |  | | | | | 12.77^***^ | | | | | 6.37^*^ | | | | | |  | | | | | | | |
| 16-year-olds |  | | | | |  | | | | |  | | | | | |  | | | | | | | |
| **Independent variables** |  | | | | |  | | | | |  | | | | | |  | | | | | | | |
| Parental attachment | -0.06 | | 0.01 | -0.26 | -7.25^***^ | -0.05 | 0.01 | -0.21 | | -5.48^***^ | -0.04 | 0.01 | -0.16 | | -4.11^***^ | |  |  | | |  | |  | |
| Social group attachment |  | |  |  |  | -1.26 | 0.25 | -0.19 | | -5.12^***^ | -1.20 | 0.24 | -0.18 | | -4.97^***^ | |  |  | | |  | |  | |
| Emotion regulation |  | |  |  |  |  |  |  | |  | -0.27 | 0.06 | -0.18 | | -4.88^***^ | |  |  | | |  | |  | |
| **Model statistics** |  | | | | |  | | | | |  | | | | | |  | | | | | | | |
| *R*^2^ | .069 | | | | | .102 | | | | | .132 | | | | | |  | | | | | | | |
| Δ*R*^2^ |  | | | | | .033 | | | | | .029 | | | | | |  | | | | | | | |
| Adj. *R*^2^ | .068 | | | | | .100 | | | | | .128 | | | | | |  | | | | | | | |
| *F* | 52.53^***^ | | | | | 40.31^***^ | | | | | 35.67^***^ | | | | | |  | | | | | | | |
| Δ*F* |  | | | | | 26.21^***^ | | | | | 23.78^***^ | | | | | |  | | | | | | | |
| 17-year-olds |  | | | | |  | | | | |  | | | | | |  | | | | | | | |
| **Independent variables** |  | | | | |  | | | | |  | | | | | |  | | | | | | | |
| Parental attachment | -0.05 | | 0.01 | -0.25 | -5.66^***^ | -0.05 | 0.01 | -0.21 | | -4.61^***^ |  |  |  | |  | |  | | |  |  | |  | |
| Emotion regulation |  | |  |  |  | -0.20 | 0.06 | -0.14 | | -3.21^**^ |  |  |  | |  | |  | | |  |  | |  | |
| **Model statistics** |  | | | | |  | | | | |  | | | | | |  | | | | | | | |
| *R*^2^ | .060 | | | | | .080 | | | | |  | | | | | |  | | | | | | | |
| Δ*R*^2^ |  | | | | | .019 | | | | |  | | | | | |  | | | | | | | |
| Adj. *R*^2^ | .059 | | | | | .076 | | | | |  | | | | | |  | | | | | | | |
| *F* | 31.99^***^ | | | | | 21.46^***^ | | | | |  | | | | | |  | | | | | | | |
| Δ*F* |  | | | | | 10.33^**^ | | | | |  | | | | | |  | | | | | | | |
| 18 or older |  | | | | |  | | | | |  | | | | | |  | | | | | | | |
| **Independent variables** |  | | | | |  | | | | |  | | | | | |  | | | | | | | |
| Emotion regulation | -0.35 | | 0.06 | -0.23 | -5.88^***^ | -0.33 | 0.06 | -0.22 | | -5.60^***^ | -0.28 | 0.06 | -0.18 | | -4.53^***^ | |  | | |  |  | |  | |
| Social group attachment |  | |  |  |  | -1.02 | 0.25 | -0.16 | | -4.09^***^ | -1.07 | 0.25 | -0.17 | | -4.28^***^ | |  | | |  |  | |  | |
| Rational decision-making |  | |  |  |  |  |  |  | |  | -0.69 | 0.22 | -0.13 | | -3.18^**^ | |  | | |  |  | |  | |
| **Model statistics** |  | | | | |  | | | | |  | | | | | |  | | | | | | | |
| *R*^2^ | .054 | | | | | .080 | | | | | .095 | | | | | |  | | | | | | | |
| Δ*R*^2^ |  | | | | | .026 | | | | | .015 | | | | | |  | | | | | | | |
| Adj. *R*^2^ | .053 | | | | | .077 | | | | | .091 | | | | | |  | | | | | | | |
| *F* | 34.56^***^ | | | | | 26.07^***^ | | | | | 21.02^***^ | | | | | |  | | | | | | | |
| Δ*F* |  | | | | | 16.69^***^ | | | | | 10.11^**^ | | | | | |  | | | | | | | |

*Note*. Parental attachment was measured using the Inventory of Parent and Peer Attachment (IPPA; Armsden & Greenberg, 1987; Portuguese version by Machado & Oliveira, 2007). Social group attachment was measured using the Social Group Attachment Scale (SGAS; Smith et al., 1999; Portuguese version by Dinis et al., 2008). Emotion regulation was measured using the Situational Test of Emotional Management-Brief (STEM-B; Allen et al., 2015; Portuguese version by da Motta et al., 2021). The unstandardized, as well as the standardized, regression coefficients are presented.

^*^*p* < .05; ^**^*p* < .01; ^***^*p* < .001

Supplementary Table 3. Pearson Correlation Coefficients between Impulsivity and Possible Negative Correlates and Protective Factors by Gender and Age Group

|  | Impulsivity | |
| --- | --- | --- |
|  | *n* | *r* |
| Younger males (13-15) |  |  |
| Early memories of warmth and safeness | 1,467 | -.07^*^ |
| Rational decision-making style | 1,061 | -.01 |
| Resilience | 1,340 | -.01 |
| Emotion regulation | 1,514 | -.16^***^ |
| Coping | 1,087 | .18^***^ |
| Parental attachment | 1,083 | -.25^***^ |
| Social group attachment | 942 | -.25^***^ |
| School satisfaction | 1,512 | -.12^***^ |
| Satisfaction with classmates | 1,512 | -.04 |
| Satisfaction with peers from other classes | 1,513 | -.01 |
| Satisfaction with friends from school | 1,513 | -.02 |
| Satisfaction with friends from outside of school | 1,511 | .01 |
| Satisfaction with teachers | 1,516 | -.13^***^ |
| Satisfaction with school staff | 1,514 | -.09^***^ |
| Satisfaction with parents | 1,512 | -.01 |
| Satisfaction with siblings | 1,410 | -.02 |
| Satisfaction with remaining family | 1,384 | .01 |
| Younger females (13-15) |  |  |
| Early memories of warmth and safeness | 1,769 | -.21^***^ |
| Rational decision-making style | 1,300 | -.14^***^ |
| Resilience | 1,612 | -.09^***^ |
| Emotion regulation | 1,815 | -.15^***^ |
| Coping | 1,309 | .10^***^ |
| Parental attachment | 1,314 | -.29^***^ |
| Social group attachment | 1,144 | -.23^***^ |
| School satisfaction | 1,811 | -.14^***^ |
| Satisfaction with classmates | 1,810 | -.06^*^ |
| Satisfaction with peers from other classes | 1,811 | -.01 |
| Satisfaction with friends from school | 1,807 | -.04 |
| Satisfaction with friends from outside of school | 1,812 | .04 |
| Satisfaction with teachers | 1,815 | -.18^***^ |
| Satisfaction with school staff | 1,815 | -.08^***^ |
| Satisfaction with parents | 1,810 | -.13^***^ |
| Satisfaction with siblings | 1,666 | -.12^***^ |
| Satisfaction with remaining family | 1,714 | -.11^***^ |
| Older males (16-19) |  |  |
| Early memories of warmth and safeness | 1,283 | -.01 |
| Rational decision-making style | 961 | -.05 |
| Resilience | 1,170 | .05 |
| Emotion regulation | 1,326 | -.21^***^ |
| Coping | 956 | .12^***^ |
| Parental attachment | 981 | -.20^***^ |
| Social group attachment | 906 | -.22^***^ |
| School satisfaction | 1,327 | -.05 |
| Satisfaction with classmates | 1,329 | -.03 |
| Satisfaction with peers from other classes | 1,326 | .03 |
| Satisfaction with friends from school | 1,327 | .02 |
| Satisfaction with friends from outside of school | 1,329 | .09^***^ |
| Satisfaction with teachers | 1,329 | -.07^*^ |
| Satisfaction with school staff | 1,327 | .01 |
| Satisfaction with parents | 1,328 | .01 |
| Satisfaction with siblings | 1,272 | .02 |
| Satisfaction with remaining family | 1,260 | -.01 |
| Older females (16-19) |  |  |
| Early memories of warmth and safeness | 1,476 | -.13^***^ |
| Rational decision-making style | 1,115 | -.16^***^ |
| Resilience | 1,338 | .02 |
| Emotion regulation | 1,505 | -.19^***^ |
| Coping | 1,133 | .07^*^ |
| Parental attachment | 1,131 | -.25^***^ |
| Social group attachment | 1,037 | -.19^***^ |
| School satisfaction | 1,496 | -.05 |
| Satisfaction with classmates | 1,504 | -.05^*^ |
| Satisfaction with peers from other classes | 1,501 | -.01 |
| Satisfaction with friends from school | 1,502 | -.02 |
| Satisfaction with friends from outside of school | 1,501 | -.02 |
| Satisfaction with teachers | 1,504 | -.09^***^ |
| Satisfaction with school staff | 1,503 | -.07^**^ |
| Satisfaction with parents | 1,505 | -.15^***^ |
| Satisfaction with siblings | 1,413 | -.09^***^ |
| Satisfaction with remaining family | 1,452 | -.08^**^ |

*Note*. Impulsivity was measured using the Impulse, Self-harm and Suicide Ideation Questionnaire for Adolescents (ISSIQ-A; Barreto Carvalho et al., 2015). Rational decision-making style was measured using the General Decision-Making Style Scale (GDMSS; Scott & Bruce, 1995; Brazilian version by Löbler et al., 2019). Resilience was measured using the Resilience Scale (RS; Wagnild & Young, 1993; Portuguese version by Felgueiras et al., 2010). Emotion regulation was measured using the Situational Test of Emotion Management-Brief (STEM-B; Allen et al., 2015; Portuguese version by da Motta et al., 2021). Coping was measured using the Toulousiana Coping Scale (TCS; Esparbès et al., 1993; Portuguese version by Amaral-Bastos et al., 2015). Parental attachment was measured using the Inventory of Parent and Peer Attachment (IPPA; Armsden & Greenberg, 1987; Portuguese version by Machado & Oliveira, 2007). Social group attachment was measured using the Social Group Attachment Scale (SGAS; Smith et al., 1999; Portuguese version by Dinis et al., 2008).

^*^*p* < .05; ^**^*p* < .01; ^***^*p* < .001

Supplementary Table 4. Results of the Stepwise Multiple Linear Regression of Impulsivity in Younger (13-15) and Older (16-19) Adolescents by Gender

|  | **Model 1** | | | | | | | **Model 2** | | | | | **Model 3** | | | | | | **Model 4** | | | | | |
| --- | --- | --- | --- | --- | --- | --- | --- | --- | --- | --- | --- | --- | --- | --- | --- | --- | --- | --- | --- | --- | --- | --- | --- | --- |
|  | *B* | | *SE* | β | | *t* | | *B* | *SE* | β | | *t* | *B* | *SE* | | β | | *t* | *B* | *SE* | | β | | *t* |
| Younger males (13-15) |  | |  |  | |  | |  |  |  | |  |  |  | |  | |  |  |  | |  | |  |
| **Independent variables** |  | |  |  | |  | |  |  |  | |  |  |  | |  | |  |  |  | |  | |  |
| Parental attachment | -0.07 | | 0.01 | -0.26 | | -7.88^***^ | | -0.06 | 0.01 | -0.20 | | -5.97^***^ | -0.05 | 0.01 | | -0.17 | | -4.91^***^ |  |  | |  | |  |
| Social group attachment |  | |  |  | |  | | -1.37 | 0.24 | -0.19 | | -5.70^***^ | -1.32 | 0.24 | | -0.19 | | -5.53^***^ |  |  | |  | |  |
| Emotion regulation |  | |  |  | |  | |  |  |  | |  | -0.21 | 0.06 | | -0.12 | | -3.68^***^ |  |  | |  | |  |
| **Model statistics** |  | | | | | | |  | | | | |  | | | | | |  | | | | | |
| *R*^2^ | .066 | | | | | | | .100 | | | | | .113 | | | | | |  | | | | | |
| Δ*R*^2^ |  | | | | | | | .033 | | | | | .014 | | | | | |  | | | | | |
| Adj. *R*^2^ | .065 | | | | | | | .098 | | | | | .010 | | | | | |  | | | | | |
| *F* | 62.10^***^ | | | | | | | 48.40^***^ | | | | | 37.23^***^ | | | | | |  | | | | | |
| Δ*F* |  | | | | | | | 32.46^***^ | | | | | 13.51^***^ | | | | | |  | | | | | |
| Younger females (13-15) |  | | | | | | |  | | | | |  | | | | | |  | | | | | |
| **Independent variables** |  | | | | | | |  | | | | |  | | | | | |  | | | | | |
| Parental attachment | -0.05 | 0.01 | | | -0.26 | | -7.62^***^ | -0.04 | 0.01 | | -0.21 | -5.81^***^ | -0.04 | 0.01 | -0.19 | | -5.12^***^ | |  |  |  | |  | |
| Social group attachment |  |  | | |  | |  | -0.90 | 0.22 | | -0.15 | -4.13^***^ | -0.89 | 0.22 | -0.15 | | -4.12^***^ | |  |  |  | |  | |
| Satisfaction with teachers |  |  | | |  | |  |  |  | |  |  | -0.65 | 0.21 | -0.11 | | -3.08^**^ | |  |  |  | |  | |
| **Model statistics** |  | | | | | | |  | | | | |  | | | | | |  | | | | | |
| *R*^2^ | .068 | | | | | | | .087 | | | | | .098 | | | | | |  | | | | | |
| Δ*R*^2^ |  | | | | | | | .019 | | | | | .011 | | | | | |  | | | | | |
| Adj. *R*^2^ | .067 | | | | | | | .085 | | | | | .095 | | | | | |  | | | | | |
| *F* | 58.05^***^ | | | | | | | 38.12^***^ | | | | | 28.85^***^ | | | | | |  | | | | | |
| Δ*F* |  | | | | | | | 17.03^***^ | | | | | 9.50^**^ | | | | | |  | | | | | |
| Older males (16-19) |  | | | | | | |  | | | | |  | | | | | |  | | | | | |
| **Independent variables** |  | | | | | | |  | | | | |  | | | | | |  | | | | | |
| Emotion regulation | -0.36 | 0.05 | | | -0.24 | | -7.21^***^ | -0.32 | 0.05 | | -0.21 | -6.46^***^ | -0.28 | 0.05 | -0.19 | | -5.55^***^ | |  |  |  | |  | |
| Social group attachment |  |  | | |  | |  | -1.29 | 0.23 | | -0.18 | -5.64^***^ | -1.12 | 0.24 | -0.16 | | -4.70^***^ | |  |  |  | |  | |
| Parental attachment |  |  | | |  | |  |  |  | |  |  | -0.02 | 0.01 | -0.09 | | -2.69^**^ | |  |  |  | |  | |
| **Model statistics** |  | | | | | | |  | | | | |  | | | | | |  | | | | | |
| *R*^2^ | .056 | | | | | | | .089 | | | | | .097 | | | | | |  | | | | | |
| Δ*R*^2^ |  | | | | | | | .033 | | | | | .008 | | | | | |  | | | | | |
| Adj. *R*^2^ | .055 | | | | | | | .087 | | | | | .094 | | | | | |  | | | | | |
| *F* | 52.03^***^ | | | | | | | 42.84^***^ | | | | | 31.19^***^ | | | | | |  | | | | | |
| Δ*F* |  | | | | | | | 31.82^***^ | | | | | 7.26^**^ | | | | | |  | | | | | |
| Older females (16-19) |  | | | | | | |  | | | | |  | | | | | |  | | | | | |
| **Independent variables** |  | | | | | | |  | | | | |  | | | | | |  | | | | | |
| Parental attachment | -0.05 | 0.01 | | | -0.26 | | -8.07^***^ | -0.04 | 0.01 | | -0.23 | -6.93^***^ | -0.04 | 0.01 | -0.20 | | -5.96^***^ | | -0.03 | 0.01 | -0.17 | | -4.79^***^ | |
| Emotion regulation |  |  | | |  | |  | -0.25 | 0.05 | | -0.17 | -5.24^***^ | -0.22 | 0.05 | -0.15 | | -4.55^***^ | | -0.21 | 0.05 | -0.15 | | -4.32^***^ | |
| Rational decision-making |  |  | | |  | |  |  |  | |  |  | -0.48 | 0.18 | -0.09 | | -2.59^*^ | | -0.53 | 0.18 | -0.10 | | -2.87^**^ | |
| Social group attachment |  |  | | |  | |  |  |  | |  |  |  |  |  | |  | | -0.55 | 0.20 | -0.10 | | -2.81^**^ | |
| **Model statistics** |  | | | | | | |  | | | | |  | | | | | |  | | | | | |
| *R*^2^ | .070 | | | | | | | .098 | | | | | .105 | | | | | | .113 | | | | | |
| Δ*R*^2^ |  | | | | | | | .028 | | | | | .007 | | | | | | .008 | | | | | |
| Adj. *R*^2^ | .069 | | | | | | | .096 | | | | | .102 | | | | | | .109 | | | | | |
| *F* | 65.15^***^ | | | | | | | 47.27^***^ | | | | | 33.95^***^ | | | | | | 27.65^***^ | | | | | |
| Δ*F* |  | | | | | | | 27.41^***^ | | | | | 6.70^*^ | | | | | | 7.92^**^ | | | | | |

*Note*. Parental attachment was measured using the Inventory of Parent and Peer Attachment (IPPA; Armsden & Greenberg, 1987; Portuguese version by Machado & Oliveira, 2007). Social group attachment was measured using the Social Group Attachment Scale (SGAS; Smith et al., 1999; Portuguese version by Dinis et al., 2008). Emotion regulation was measured using the Situational Test of Emotional Management-Brief (STEM-B; Allen et al., 2015; Portuguese version by da Motta et al., 2021). The unstandardized, as well as the standardized, regression coefficients are presented.

^*^*p* < .05; ^**^*p* < .01; ^***^*p* < .001
